# A Combination of Divergence and Conservatism in the Niche Evolution of the Moorish Gecko, *Tarentola mauritanica* (Gekkota: Phyllodactylidae)

**DOI:** 10.1371/journal.pone.0127980

**Published:** 2015-05-22

**Authors:** Catarina Rato, David James Harris, Ana Perera, Silvia B. Carvalho, Miguel A. Carretero, Dennis Rödder

**Affiliations:** 1 CIBIO Research Centre in Biodiversity and Genetic Resources, InBIO, Universidade do Porto, Campus Agrário de Vairão, Rua Padre Armando Quintas, Vairão, Vila do Conde, Portugal; 2 Zoologisches Forschungsmuseum Alexander Koenig, Bonn, Germany; Trier University, GERMANY

## Abstract

The quantification of realized niche overlap and the integration of species distribution models (SDMs) with calibrated phylogenies to study niche evolution are becoming not only powerful tools to understand speciation events, but can also be used as proxies regarding the delimitation of cryptic species. We applied these techniques in order to unravel how the fundamental niche evolved during cladogenesis within the *Tarentola mauritanica* species-complex. Our results suggest that diversification within this complex, during the Miocene and Pleistocene, is associated with both niche divergence and niche conservatism, with a pattern that varies depending on whether the variables involved are related to the mean or seasonality of temperature and humidity. Moreover, climatic variables related to humidity and temperature seasonality were involved in the niche shift and genetic diversification of the European/North African clade during the Pleistocene and in its maintenance in a fundamental niche distinct from that of the remaining members of the group. This study further highlights the need for a taxonomic revision of the *T*. *mauritanica* species-complex.

## Introduction

**Fig 1:**
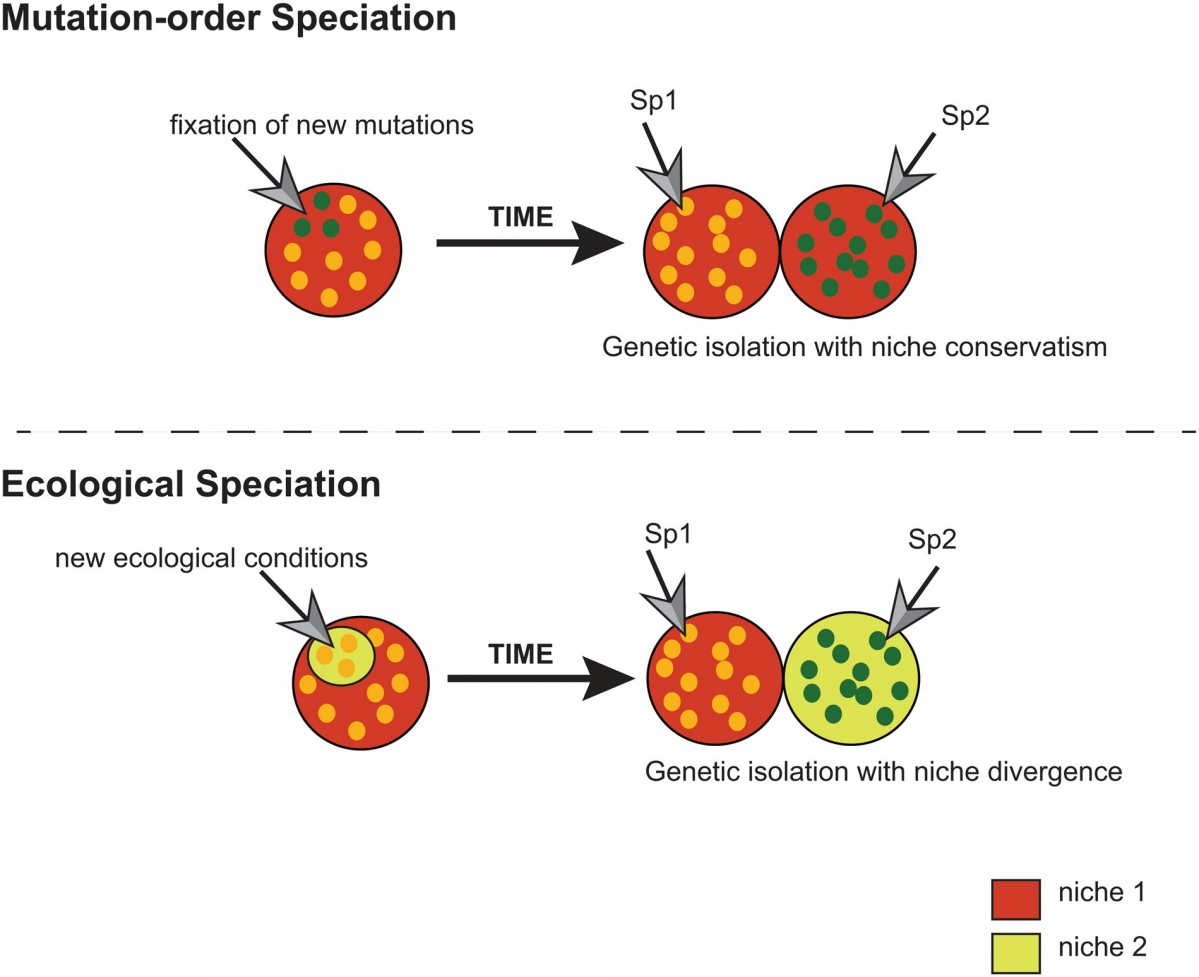
Illustration of mutation-order and ecological speciation. In mutation-order speciation, there is the initial fixation of advantageous mutations in the population, which will increase in frequency, leading to the genetic isolation of species in the same ecological niche. Ecological speciation occurs when new environmental conditions appear in the population with the adaptation of some individuals to this novel state and divergence of their niche and consequent genetic diversification from the original population.

Speciation by natural selection occurs mainly through two distinct mechanisms; mutation-order and ecological speciation [1,2,3] (Fig 1). Mutation-order speciation occurs when distinct advantageous or neutral mutations are fixed by chance between different populations/entities, while these are under similar ecological conditions or selective pressures [1,3]. The fixation of such mutations by drift, reduces the fitness of hybrids over evolutionary time scales [4]. On the other hand, ecological speciation refers to the evolution of reproductive isolation between species primarily by differential adaptation to distinct environmental or ecological conditions [5,6,7]. In this context, natural selection acts as a divergent mechanism driving to fixation distinct advantageous mutations in each of the different environments [1,2]. Whether ecological divergence may constitute the primary force inducing speciation or simply acts secondarily after an interruption of gene flow due to other causes may be difficult to ascertain in practice by monitoring the ecological traits of clades in a given phylogeny [1]. However, the appraisal of ecological divergence can sometimes serve as a proxy to support species delimitation to understand which ecologically associated factors led to speciation (e.g. [8,9,10,11,12]).

During ecological divergence, natural populations are subjected to different biotic and abiotic factors, such as predation, competition, climate and food resource fluctuations [1,5], which may lead to distinct evolutionary responses and patterns of climatic tolerances (reviewed in [13]) and to the consequent evolution of organisms in new habitats (niche divergence) (e.g. [8,14,15,16,17]). On the contrary, niche conservatism consists of the preservation of ecological similarity among populations over time [18,19], being important in the classic model of allopatric speciation, as it may limit adaptation to the ecological conditions at the geographic barrier and promote genetic isolation and differentiation between vicariant populations (e.g. [15,16,20,21]) (Fig 1). Hence, the assessment of whether the realized niche of natural populations has evolved in a conservative [18,19,20,22] or divergent manner [1,2,5,23] is of paramount importance, in order to evaluate the possibility of an ecological speciation event having occurred.

Not surprisingly, the study of niche evolution has increased in popularity in the last few years, focusing on techniques to estimate overlap in realized niches of different species in an explicit spatial context [24], either based on ordination methods [25,26] or on the output of species distribution models (SDMs) [27,28]. More recently, based on the methodology proposed by Evans et al. [29], SDMs are being combined with calibrated phylogenies in order to study the evolution of realized niches by reconstructing the ancestral environmental tolerances among clades (e.g. [30,31,32]).

**Fig 2:**
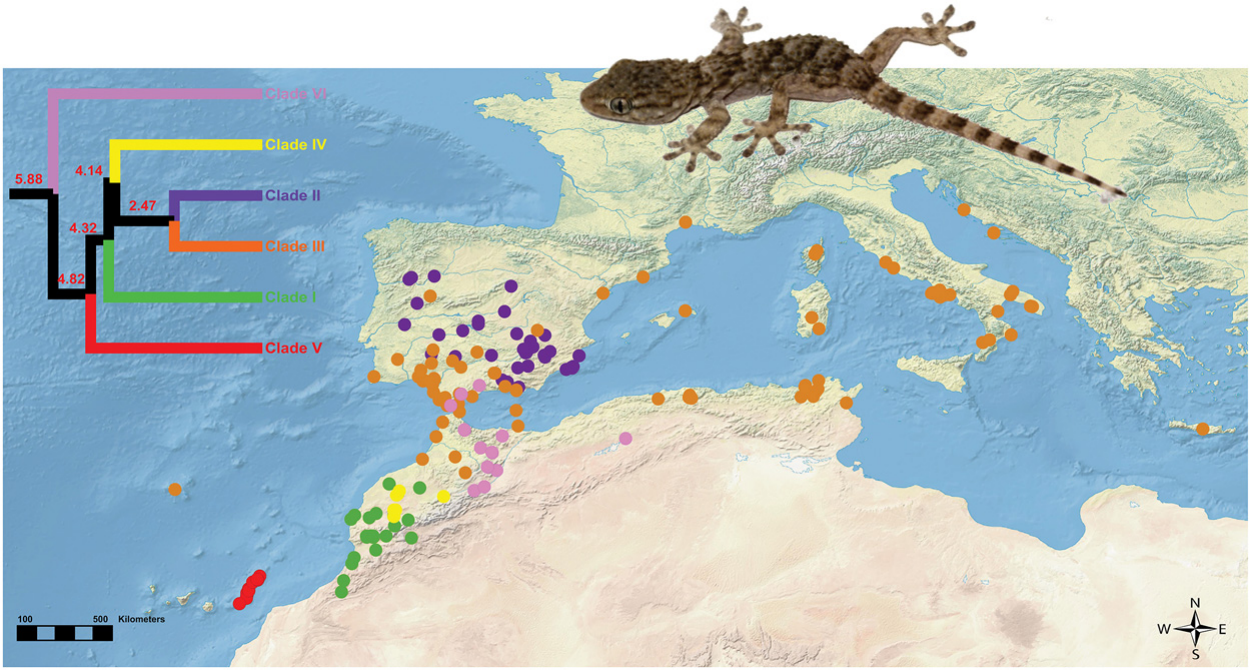
Map illustrating the occurrence records of all six clades of *Tarentola mauritanica* used for the SDMs and a calibrated phylogenetic tree with node ages (in Ma), modified from Rato et al. [34]. The tree was obtained using concatenated nuclear (ACM4, MC1R, PDC, and RAG2) and mtDNA (12S rRNA and 16S rRNA) loci. Calibration was performed with the software BEAST [87], considering the mean substitution rates and the standard error of the mean values for 12S, extracted from a fully calibrated phylogeny of *Tarentola* from the Canary Islands [70,88].

The Moorish gecko, *Tarentola mauritanica* (Linnaeus, 1758), is a species-complex distributed across the Mediterranean Basin, comprising six mitochondrial evolutionary clades, with well-defined geographic ranges (Fig 2); Clade I is present in Central and Southwestern Morocco; Clade II is exclusively Iberian [33]; Clade III is present in Southern Europe and North Africa; Clade IV occupies Central Morocco; *T*. *angustimentalis*, endemic to the Eastern Canary Islands (Clade V) lies within the *T*. *mauritanica* complex; and, finally, Clade VI is distributed across Southern Iberia, Northeastern Morocco and Northwestern Algeria [33,34,35,36,37]. A recent study by Rato et al. [34] estimated that the oldest cladogenesis event within this group occurred around 5.88 Mya while the most recent one took place 2.47 Mya [34] (Fig 2). These six groups appear to represent several closely related cryptic clades exhibiting high levels of mtDNA diversity, but with nuclear phylogenies demonstrating limited evidence of monophyly due to incomplete lineage sorting [34,37]. However, the paraphyly of *T*. *mauritanica*, as currently recognized, together with the obtained high levels of mtDNA divergence, indicate that a taxonomic revision is needed [34]. Surprisingly, low levels of mtDNA variation within the European/North African clade have been observed, when compared to the others. This was initially attributed to a recent colonization [33,36,37], particularly given the frequency with which *T*. *mauritanica* is associated with humanized environments, leading to anthropogenic introductions [38]. However, recent molecular investigation of this pattern revealed that nuclear loci do not show this lack of variation and, in fact, they exhibit higher nucleotide diversity values compared to the used mtDNA markers [39]. This is consistent to a process where selection (most likely positive selection) is acting only on the mitochondrial variation of this clade, increasing the frequency of one allele and causing a drop in the overall variability for this marker [39,40]. Similar findings have been reported for pikas [41], monkeys [42] and even teleost fishes [43], where positive selection of the mtDNA has led to an increased capacity to inhabit environments characterized by low temperatures. A similar scenario could be hypothesized for the European/North African clade, which occurs in colder environments. In this study, we aim to: (1) evaluate if fundamental niche evolution is concordant with the currently accepted clades; (2) assess the degree to which phylogenetic proximity predicts niche conservatism (i.e. similarity in environmental tolerances); and (3) test if there is a climatic tolerance divergence of the European/North African clade, with respect to the remaining ones.

To achieve these goals, we used ordination methods and SDMs to estimate niche differences and similarities between the clades of *T*. *mauritanica*. Subsequently, the evolutionary history of niche occupancy of each clade of *T*. *mauritanica* was reconstructed by merging the SDMs with a published calibrated multilocus phylogeny of the genus *Tarentola* [34].

## Materials and Methods

### Environmental variables

In order to quantify the realized niches of the different clades of *Tarentola mauritanica* at a geographic scale, we computed a set of multi-temporal variables capturing major annual changes in environmental conditions based on pre-processed monthly variables derived from the MODIS sensors of two NASA satellites and available at http://www.edenextdata.com. The spatial and temporal resolution of the data sets are 30 arc sec for both, 8-day averages for MOD11A2 and 16-day averages for MCD43B4 (for more details regarding sensor products and variables extraction see [44,45]), covering the period 2001–2005. In this study, we used five types of variables, each represented by monthly grids capturing averages, namely Middle Infra-Red (MIR), Daytime (DTLST) and Night Time (NTLST) Land Surface Temperature, Normalized Difference Vegetation Index (NDVI) [46] and Enhanced Vegetation Index (EVI) [47] totalling 60 single GIS layers (5 types of variables x 12 monthly variables). These 60 monthly variables were then transformed into bioclimatic variables to capture major annual seasonal variations using the *dismo* (i.e. *biovars* function) and *raster* packages [48,49] for Cran R 2.15 [50] (for details see S1 Table). NDVI allows the assessment of whether the target contains live green vegetation, barren areas of rock, and or snow. While the EVI is calculated similarly to NDVI, it corrects for some distortions in the reflected light caused by the particles in the air as well as the ground cover below the vegetation. Since in most climates, vegetation growth is limited by water, rather than vegetation per se these two indices are here used as proxies for the availability of humidity in the system (http://earthobservatory.nasa.gov/Features/MeasuringVegetation/). Analogous to the 19 original bioclimatic variables (from http://www.worldclim.org/bioclim) capturing seasonal extremes and variations in temperature and precipitation patterns as well as interactions among both, we computed a set of variables capturing annual variations based on the remote sensing variables. For temperature related variables, the respective subset of bioclimatic variables was derived from monthly average daytime and nighttime land surface temperatures (i.e: Annual Mean Temperature; Mean Diurnal Range; Isothermality; Temperature Seasonality; Maximum Temperature of Warmest Month; Minimum Temperature of Coldest Month; Temperature Annual Range). For variables describing the vegetation structure (NDVI and EVI; [51]) and proxy for humidity (MIR), only the respective set of bioclimatic temperature variables were computed. The final set of variables was clipped to the general extent of the species’ distribution. Multi-co-linearity of the 30 predictors was removed by performing a principal component analysis (PCA) computed in R [50] summarizing the environmental conditions, in which only those principal components with eigenvalues > 1 were retained. For detailed information on the workflow to create the 30 variables, see legend from S1 Table.

### Species distribution models (SDMs)

Occurrence records of the animals from the different clades used to train the SDMs were the same as in Rato et al. [34], plus six additional specimens of *T*. *angustimentalis* from the Canary Islands (Fuerteventura and Lanzarote) collected in October 2012, used only in our study (see S2 Table for geographic coordinates and clade assignment). Experienced herpetologists carried out the collection and handling of the specimens captured in both Canary Islands. After identification of the species and recording the GPS coordinates, all animals were released at the site of capture and none of them was sacrificed. This protocol has been approved by the Committee of Animal Experimentation of the University of Porto (Portugal) under the Directive 2010/63/EU of the European Parliament. All captures were carried out in public land and authorized by the environmental authorities from both islands (Consejera de Pesca, Caza, Ambiente Ambiente y Aula de la Naturaleza del Cabildo de Lanzarote and Consejería de Medio Ambiente del Cabildo de Fuerteventura) using the permits 12570 and 2932, respectively. These permits were issued specifically for our study.

All records correspond to locations of specimens collected in the field and genetically assessed by Rato et al. [34]. The final dataset comprised 261 locations, corresponding to 40, 47, 121, 15, 21 and 17 records from clades I through VI, respectively. All records are illustrated in Fig 2.

The realized niches of the different clades were calculated by computing boxcar environmental envelopes, using the Bioclim algorithm [52] as implemented in the *dismo* package [48] for R [50]. Principal Components obtained previously from the environmental variables listed in S1 Table were used as input data. We preferred to apply a rather simple algorithm here, such as Bioclim, since it is best suited to summarize the realized niches of the clades without taking the explanatory power of the variables into account, nor their interactions [53]. This may more closely reflect Hutchinson’s [54] original idea of a species’ Grinnellian niche which constitutes all environmental conditions under which populations may exist, irrespective of any weighting of variables.

### Realized niche overlap analyses

Based on the environmental variables X1–X30 listed in S1 Table, we computed univariate niche density plots using the *sm* package [55] for R [50], as well as pairwise niche overlaps and both niche background similarity and niche equivalency tests *sensu* Warren et al. [28] among clades. Computations were conducted applying a statistical framework termed PCA-env, which was recently proposed by Broennimann et al. [24]. In this framework, the available environmental space of two clades, as defined by all conditions within a buffer of 100 km enclosing the species records representing the potentially colonisable environmental space (see also [56]) accounting for both natural and human mediated dispersal, was used to train a PCA. Both set of species records and each set of available environmental conditions were projected into this PCA space. Subsequently, the relative density of background conditions and species records across the first two PCs were captured by a kernel density smoother in order to create density grids of *r* x *r* cells in environmental space. We set the resolution of *r* to 100. These density grids were used to compute niche overlaps in terms of Schoener´s *D* (as reviewed in [28,57]), which seems to perform better than other metrics [58]. Schoener´s *D* ranges from 0 (no overlap) to 1 (complete overlap).

Hypotheses of niche equivalency and niche similarity *sensu* Warren et al. [28] were computed from the density estimations of species in environmental space following Broennimann et al. [24]. The niche equivalency test determines whether niches of two entities in two geographical ranges are effectively more equivalent than expected by chance, whereas the niche similarity test addresses whether the realized niche overlap among entities can be attributed to the available environmental space of one entity or to active habitat selection. For the niche equivalency test, all occurrences of the two entities were pooled and randomly split into two datasets, repeated 100 times, and the niche overlap statistic *D* was calculated. These simulated values were used to construct 95% confidence intervals (CIs). If the observed value of niche overlap falls within these CIs, the null hypothesis of niche equivalency cannot be rejected. For the niche similarity test, niche overlaps are computed based on the environmental conditions at the records of one taxon and randomly generated records within the available environmental space of the second taxon. Therefore, this test is computed in both directions assessing whether the observed niche overlap can be attributed to the available environmental spaces. If the observed overlap is greater or smaller than 95% of the simulated values, the null hypothesis is rejected indicating that niche differentiation between entities is derived by habitat selection and is not an artefact related to differences of the underlying environment. Otherwise, it is not possible to distinguish between overlaps due to active habitat selection or availability of specific environmental conditions. All computations were conducted in R [50] using the scripts provided by Broennimann et al. [24], which were adapted to our dataset.

### History of niche occupancy

To reconstruct the ancestral niche occupancy by each of the six clades of *T*. *mauritanica*, we combined all of their potential distributions (SDMs) with a calibrated ultrametric tree modified from the original multilocus phylogeny published by Rato et al. [34]; the ultrametric tree used here corresponds to a subtree of the original, including only the clades of interest, calculated using the software DENDROSCOPE v.1.2.4 [59]; the branches of each clade were collapsed to a single terminal, using the *drop*.*tip* function from the R package *ape* [60]. Predicted niche occupancy (PNO) profiles were generated following Evans et al. [29] as implemented in the *phyloclim* package [61] for R. For each clade and corresponding PCs scores, the probability distribution derived from Bioclim was binned into 100 evenly spaced categories, in order to obtain a PNO profile per PC. From these PNO profiles, we drew 1000 random samples to estimate the climatic tolerances of ancestral nodes, assuming a Brownian motion evolution and using the generalized least squares method [62,63]. Ambiguities of relationships between clades were taken into account by repeating the analysis over 1000 samples from the posterior distribution of the ultrametric tree [29,64].

## Results

### Species distribution models (SDMs)

The dimensionality of the 30 variables listed in S1 Table was reduced into four principal components, with the first two explaining 83.15% of the total variance (PC1 explaining 71% and PC2 12% of the total variance). As for PC1, most of the variation along the axis was explained by 21 variables (|r| ≥ 0.8) pertaining to different temporal transformations of humidity (Middle Infra-Red), Land Surface Temperature, Normalised Difference Vegetation Index (NDVI) and Enhanced Vegetation Index (EVI), while PC2 was mainly associated with seasonality (X4) and annual range (X7) of the Middle Infra-Red and by the seasonality of the Land Surface Temperature (X13), (for details see S1 Table).

**Fig 3:**
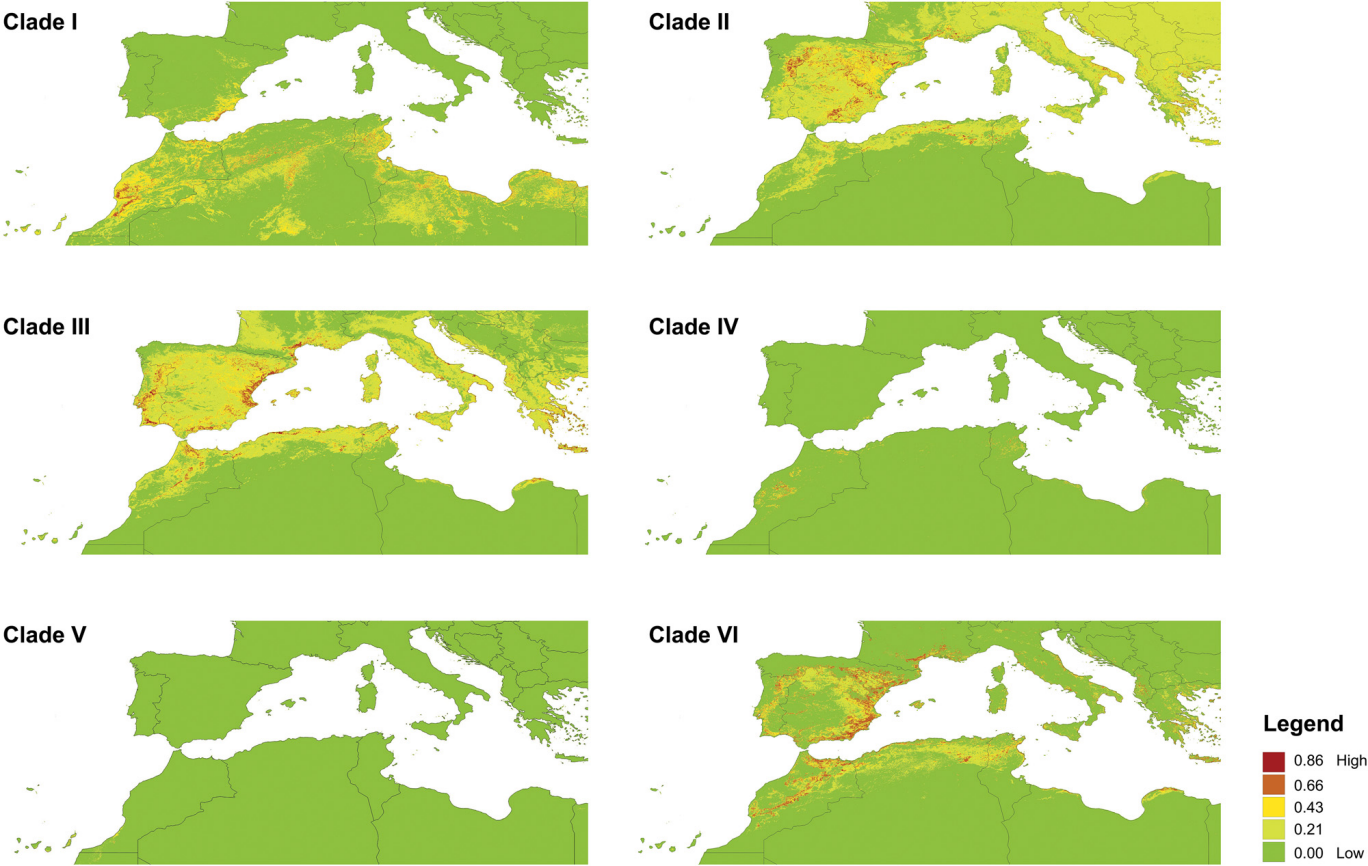
Species distribution models (SDMs) for the six clades of *Tarentola mauritanica*. Warmer colours indicate higher environmental suitability.

Visual inspection of the potential occurrence of each clade in space (Fig 3) showed that for some of them, the areas of high probability of occurrence are quite similar to their currently known distribution (Clades III and V; see details in Fig 2). In contrast, for the South/Central Moroccan clade (Clade I), the Iberian (Clade II), the Central Moroccan clade (Clade IV) and the North African Southern Iberian clade (Clade VI), the SDMs predicted a much larger area of potential occurrence. Specifically, for Clade I suitable conditions occur in Northern Maghreb and the Southeastern portion of the Iberian Peninsula; Clade II potentially ranged from the Iberian Peninsula all the way to the Balkans and Northern Maghreb; Clade IV also potentially included the Northern border between Morocco and Algeria and Northeastern Tunisia; and Clade VI potentially covered the entire Western Mediterranean region. It is noteworthy that the European/North African clade (Clade III) was mostly restricted to Mediterranean environments, being predicted to occur primarily near the coast. The same holds true for Clade VI.

### Realized niche overlap analyses

Results from PCA-env analyses demonstrated that for all pairwise comparisons among clades, the sum of the first two axes of the PCA explained between 65.97% and 77.05% of the total variance. This information, as well as the contribution of the 30 variables to each PC for all pairs of clades compared, is illustrated in more detail in S1 Fig. Regarding the pairwise assessment of niche overlap using the metric *D* [57] (Table 1), this measure varied between 0.03 (Clade III—Clade IV) and 0.43 (Clade III—Clade VI), corresponding to indices of no or very limited overlap and moderate overlap, respectively, according to the metrics suggested by Rödder and Engler [58]. For the pairs I-II, I-III, I-VI, II-VI, II-VI and IV-V, niche overlap is higher than expected based on the environmental conditions available to each but significant results were obtained in only one direction.

**Table 1:** Niche overlap analyses.

Comparison (1–2)	Niche overlap (*D*)	Niche Equivalency	Niche Similarity (2→1)	Niche Similarity (1→2)
Clade I—Clade II	0.26 (low)	**0.02**	0.06	**0.02**
Clade I—Clade III	0.32 (low)	**0.02**	**0.04**	0.99
Clade I—Clade IV	0.12 (none)	**0.02**	0.59	0.16
Clade I—Clade V	0.09 (none)	**0.02**	0.55	0.40
Clade I—Clade VI	0.26 (low)	**0.02**	0.14	**0.02**
Clade II—Clade III	0.33 (low)	**0.02**	**0.02**	**0.02**
Clade II—Clade IV	0.05 (none)	**0.02**	0.57	0.32
Clade II—Clade V	0.14 (none)	**0.02**	0.20	0.44
Clade II—Clade VI	0.24 (low)	**0.02**	**0.02**	0.08
Clade III—Clade IV	0.03 (none)	**0.02**	**0.04**	**0.02**
Clade III—Clade V	0.13 (none)	**0.02**	0.08	0.61
Clade III—Clade VI	0.43 (moderate)	**0.02**	**0.02**	**0.02**
Clade IV—Clade V	0.13 (none)	**0.02**	**0.02**	0.50
Clade IV—Clade VI	0.09 (none)	**0.02**	**0.02**	0.89
Clade V—Clade VI	0.07 (none)	**0.02**	0.40	0.24

Pairwise niche overlap values using the metric *D* [57], corresponding overlap classification according to Rödder & Engler [58] and *P*-values of niche similarity and equivalence via randomization test. Significant values with *P*<0.05 in the niche equivalency test and falling outside of the 95% CI (0.025–0.975) of the niche similarity test are shown in bold.

In agreement with these relatively low values of the Schoener´s *D* metric for most pairs of clades, the niche equivalence hypothesis was rejected for all pairwise comparisons (in all cases *P*<0.05, Table 1), suggesting that all clade pairs possess more significantly distinct realized niches than expected by chance.

### History of niche occupancy

**Fig 4:**
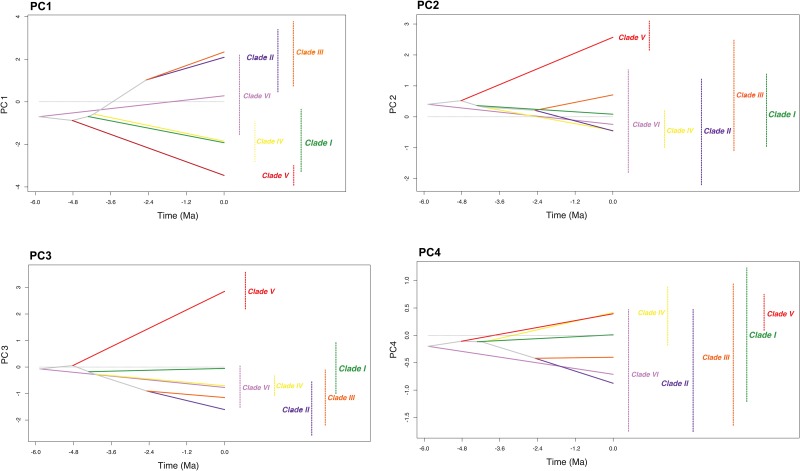
Inferred history of the evolution of climatic tolerances within the *Tarentola mauritanica* species-complex. The calibrated phylogenetic tree used, was modified from Rato et al. [34]. Interior nodes represent the mean of climatic tolerances inferred for the most recent common ancestor of the extant clades defined by that node. Solid lines connect ancestors with their descendants. A vertical dashed line indicates the 80% central density of climatic tolerance for each extant taxon, and the taxon label, to the right of each graph, indicates the mean. Lines and labels are coloured according to clades defined in Fig 2.

Results of the ancestral niche occupancy profiles (Fig 4) showed evidence of cladogenesis associated with both niche divergence and conservatism.

Niche divergence was most noticeable for Clade V, and particularly for climatic variables associated with PC1, PC2 and PC3, while niche conservatism was mainly observed between clades I and IV and clades II and III along PC1. Surprisingly, this clear evidence of ancestral niche divergence for *T*. *angustimentalis* (Clade V) is not in agreement with the results obtained for the niche overlap in Table 1. For PC1 (which explains 71% of environmental variance), the closely related clades II and III exhibited highly similar environmental affinities, as well as clades I and IV, while clades V and VI showed evidences of niche divergence from the remaining clades, although their current environmental tolerances exhibit a high level of overlap with Clade I, and clades II and III, respectively. Most of these results are in agreement with the ones obtained for the niche overlap analyses in Table 1, as most clades displayed some levels of niche overlap, except the pairs III-IV and IV-VI. PC2 predicts a slight ancestral niche divergence of the mean tolerances of Clade III from the remaining groups, although its current tolerance range still largely overlaps. Since humidity and temperature seasonality were the variables most strongly correlated with PC2, these had the highest influence on the niche tolerance pattern exhibited by all clades.

## Discussion

The study of niche evolution is of paramount importance in order to understand the speciation mechanisms behind the divergence of species. Specifically, the evaluation of whether the realized niche of natural populations has evolved in a conservative or divergent way, allows us to infer if an ecological speciation event may have taken place.

The moderate overlap value observed for the Clade III—Clade VI comparison is related to the fact that individuals from both the European/North Africa (Clade III) and the North Africa/Southern Iberian (Clade VI) clades are mostly distributed along Mediterranean climate areas (Fig 2) and, therefore, it is reasonable that their potential niches tend to overlap. The moderate niche overlap of these two clades is also supported by the results obtained with the niche similarity tests, as this comparison, along with the pair composed by clades II and III, were the only ones where a two-way significantly higher similarity than expected based on their available environmental conditions was found. Nevertheless, some authors have argued that comparison of SDMs is probably not the best surrogate for a comparison of environmental requirements (reviewed in [65]). Not surprisingly, the results obtained from this test differ from some of the niche similarity tests. For example, for clades III-IV and IV-VI niche similarity was significantly smaller than expected based on the environmental conditions available to each clade.

From an overall inspection of the environmental tolerance profiles by PC1 and PC2, the general speciation pattern within the *Tarentola mauritanica* complex, seems to have led to both niche divergence and conservatism. Niche divergence seems to be clear for Clade V, while conservatism is noticeable in clades I and IV (in both PC1 and PC2) and between clades II and III (in PC1 but not PC2). Furthermore, there is no evidence of similar environmental tolerances with phylogenetic proximity (except for clades II and III in PC1). However, when analysing the results obtained for the niche overlap analyses, it is apparent that currently some of the clades occupy quite similar ecological conditions.

Around 5.88 Mya, the North African/Southern Iberian Clade (Clade VI) became separated from the remaining clades, matching very closely the closing of the Strait of Gibraltar, and the Messinian Salinity Crisis (5.959 to 5.33 Mya) [66,67,68]. Since there are no fossil records of *Tarentola* in the Iberian Peninsula from this period [69], we can only assume that individuals did not cross from North Africa to Europe during the Miocene. Results from the niche overlap analyses confirm that Clade VI still occupies a relatively distinct realized niche from Clade IV and the ancestral climatic tolerance profile suggests cladogenesis by ecological divergence, most probably in North Africa.

Later, the separation of the ancestor of *Tarentola angustimentalis* (Clade V) took place, when these reached the Eastern Canary Islands (Lanzarote and Fuerteventura) around 4.82 Mya, by transmarine dispersal [34,70]. In this new environment, the ancestors of *T*. *angustimentalis* were subject to novel environmental conditions, leading to their ecological divergence in allopatry with respect to their continental relatives, as suggested by their ancestral climatic tolerances. Studies on the paleoclimatology of the Canaries indicate that during the Mio-Pliocene these islands had a tropical climate similar to the present day conditions along the coast of the Gulf of Guinea and Caribbean Sea [71]. Currently, due to their old geological ages determining the erosion of the relief, Lanzarote and Fuerteventura are no longer able to retain precipitation brought by trade winds [72]. In consequence, the climate of both islands has become heavily influenced by the North African Saharan winds, presenting annual temperatures ranging from 17–25°C and 22–35°C, respectively and very low levels of precipitation per year (147 mm in Fuerteventura) [73]. Hence, it was expected that individuals of *T*. *angustimentalis* would inhabit completely distinct ecological conditions, yet the acceptance of the null hypothesis of niche background similarity test suggests that the low levels of niche overlap between this clade and the others is most likely a result of the available background of Clade V; when defining a buffer of 100 km around the species records, mostly ocean will be selected and the background will consist mainly of unsuitable environment, making it difficult to determine whether there is active niche search or not.

Around 4.32 Mya, the South-Central Moroccan clade (Clade I) split from the remaining elements, as did later the Central Moroccan clade (Clade IV), approximately 4.14 Mya. However, these clades conserved the same niche and currently show no signs of niche divergence between them. The most probable hypothesis to explain this pattern is that both clades evolved in allopatry, likely caused by vicariant barriers, resulting in a high-level of niche conservatism. This would imply that in the past their distribution was distinct from today, since they are presently in contact. Unfortunately, past SDMs can only be produced for the available palaeoscenarios, namely up to the Last inter-glacial (~120.000–140.000 years BP) [74], making it difficult to validate this hypothesis.

By contrast, the ancestor of clades II (Iberian Clade) and III (European/North African Clade) split from Clade IV, approximately 4.14 Mya, with a strong niche divergence between them. Such divergence mainly concerned the mean temperature and humidity of the areas occupied (PC1). Subsequently, around 2.47 Mya, Clade III diverged from Clade II (and from the remaining clades) occurring in areas undergoing distinct humidity-related and temperature seasonality conditions (PC2). We interpret this as an ecological shift of *Tarentola* geckos from arid to Mediterranean habitats, which indeed started to spread during the Miocene-Pliocene transition [75], and subsequent adaptation to Mediterranean continental climate of Central Iberian Peninsula. Whether the first transition took place in North Africa or in the Iberian Peninsula is debatable but certainly the second transition should have been exclusively Iberian [34], with Clade II diverging from Clade III in allopatry due to the Mediterranean acting as a barrier. Depending on the contribution of the geographic barriers (i.e. the Gibraltar Strait), such niche shifts may have played a substantial role in the speciation processes of this group, as well as allowing it to occupy more northerly regions in Europe that would have been previously unavailable. Indeed, a recent ecophysiological study comparing multiple Iberian populations belonging to these two clades [76] reports clear differences in water loss rates. Overall, the European lineage displayed a trend for higher water loss when compared to the Iberian lineage. The lack of correspondence between ecophysiological traits and local climatic conditions, support the existence of a phylogenetic signal rather than local adaptation. These results suggest that divergent evolutionary responses to the environment in both lineages, mainly acting on water ecology, may account for the differences in their range expansion [76]. These findings reinforce the evidence that humidity-related conditions led to the divergence between these two clades, at least in the Iberian Peninsula. Nonetheless, such a shift may have permitted both clades to cope with the successive expansions and retractions during the Pleistocenic climatic oscillations [77,78] and, more recently, allowed Clade III to colonize (through human-mediation) other continental and insular regions of the Mediterranean Basin [34].

Interestingly, *Tarentola* responded to the environmental changes during the Miocene resulting in a combination of niche conservatism under unchanged conditions and niche shift to newly arisen habitats (e.g. [79]). By contrast, responses to the faster climate oscillations during the Pleistocene and the Holocene are dominated by niche conservatism suggesting a degree of phylogenetic inertia (e.g. clades II and III and clades I and IV for PC1). During the glaciations, *Tarentola* populations probably survived in Iberia in various isolated “microclimate pockets” [80], which is in agreement with the “refugia-within-refugia” model, hypothesized for the Iberian Peninsula (but see [81], e.g. [82]). It is noteworthy that Clade III, the most recently divergent in terms of realized niche, shows evidence of selection, which led to the existence of a single mtDNA variant [39]. Support that individuals from this clade have chosen unique environmental conditions is corroborated not only by its ancestral niche occupancy profile in PC2, but also by the fact that none of the other clades were ever found in the range occupied by the European/North Africa clade. Taking into account that individuals of *Tarentola* are frequently associated with humanized habitats, and their accidental introductions are common [38], especially around the Mediterranean, we would expect other clades to be introduced in Europe as well. Competitive interactions between clades (e.g. [83]) can also be hypothesized, and this should be validated with extensive fieldwork and modelling in the contact areas [84] as well as by experimental tests [85].

## Conclusions

The integration of spatial ecological models with phylogenies provides a very powerful tool, helping in the delineation of cryptic species [8,9,10], or to at least giving additional support to their specific status [86]. According to the results from this study, the diversification of the clades within the *Tarentola mauritanica* species-complex fits both the niche divergence and conservatism profiles, with individuals of some clades still maintaining a realized niche distinct from their relatives. These results, allied with the high levels of mitochondrial divergence observed between clades [34], provide additional evidence for the need of a taxonomic revision of the whole complex, using a battery of nuclear markers to test species boundaries. Likewise, the niche evolution study and overlap patterns of the European/North African clade corroborate that climatic variables related to humidity and temperature seasonality (and hence, natural selection) resulted in the divergence of this clade and to the maintenance of the individuals in a realized niche distinct from the remaining members of *Tarentola mauritanica*.

## Supporting Information

S1 Fig Pairwise comparison of niches in climatic space (PCA-env) between all clades of the *Tarentola mauritanica* species-complex. Upper left and upper right plots represent the niches of the two clades compared; density of occurrence is indicated by the degree of grey-shading; solid and dashed contour lines illustrate 100% and 50% of the available environmental space, respectively. Lower left plot illustrates the contribution of each of the remote sensing variables (X1 to X30; for abbreviations see S1 Table) on the two axes of the PCA and the explanatory power of the two main axes. Red diamonds on the right graphs indicate the position of the observed niche overlap.
(PDF)

S1 Table Remote sensing variables and corresponding abbreviations used in this study. The results of the Principal Components analysis (PCA) include the component loadings and the correlation scores (in parenthesis) calculated using Pearson’s r correlation, for the first four principal components (PC). In both PC1 and PC2 the numbers in bold correspond to the variables that contributed more to each axis (absolute Pearson’s r correlation, |r| ≥ 0.8). The workflow to create the 30 variables was as following: the starting point were 5 sets of variables, each with 12 months = 60 single variables representing monthly means (12 x NLST; 12 x DLST; 12 x MIR; 12 x NDVI; 12 x EVI). For each of the set of 12 monthly variables of MIR, NDVI and EVI the following 7 temporal transformations were computed (= 21 new variables): BIO1 = Annual Mean; BIO4 = Seasonality; BIO5 = Max of Month with highest scores; BIO6 = Min of Month with lowest scores; BIO7 = Annual Range; BIO10 = Mean of Quarter with highest scores; BIO11 = Mean of the Quarter with lowest scores. Based on monthly variables of NLST and DLST the following 9 temporal transformations were computed (= 9 new variables): BIO1 = Annual Mean; BIO2 = Mean Diurnal Range; BIO3 = Isothermality; BIO4 = Seasonality; BIO5 = Max of Month with highest scores; BIO6 = Min of Month with lowest scores; BIO7 = Annual Range; BIO10 = Mean of Quarter with highest scores; BIO11 = Mean of the Quarter with lowest scores. These 30 new variables were subject to a PCA.
(DOC)

S2 Table Specimens used in this study. Individuals and their corresponding clade assignation and geographic coordinates.
(DOC)
