# Supplementary material for: A Combination of Divergence and Conservatism in the Niche Evolution of the Moorish Gecko, Tarentola mauritanica (Gekkota: Phyllodactylidae)
Source: PLoS One. 2015 May 22;10(5):e0127980. doi: 10.1371/journal.pone.0127980 (PMC4441378; doi:10.1371/journal.pone.0127980)
Supplement: S1 Fig — Upper left and upper right plots represent the niches of the two clades compared; density of occurrence is indicated by the degree of grey-shading; solid and dashed contour lines illustrate 100% and 50% of the available environmental space, respectively. Lower left plot illustrates the contribution of each of the remote sensing variables (X1 to X30; for abbreviations see S1 Table) on the two axes of the PCA and the explanatory power of the two main axes. Red diamonds on the right graphs indicate the position of the observed niche overlap. (PDF) [file pone.0127980.s001.pdf]

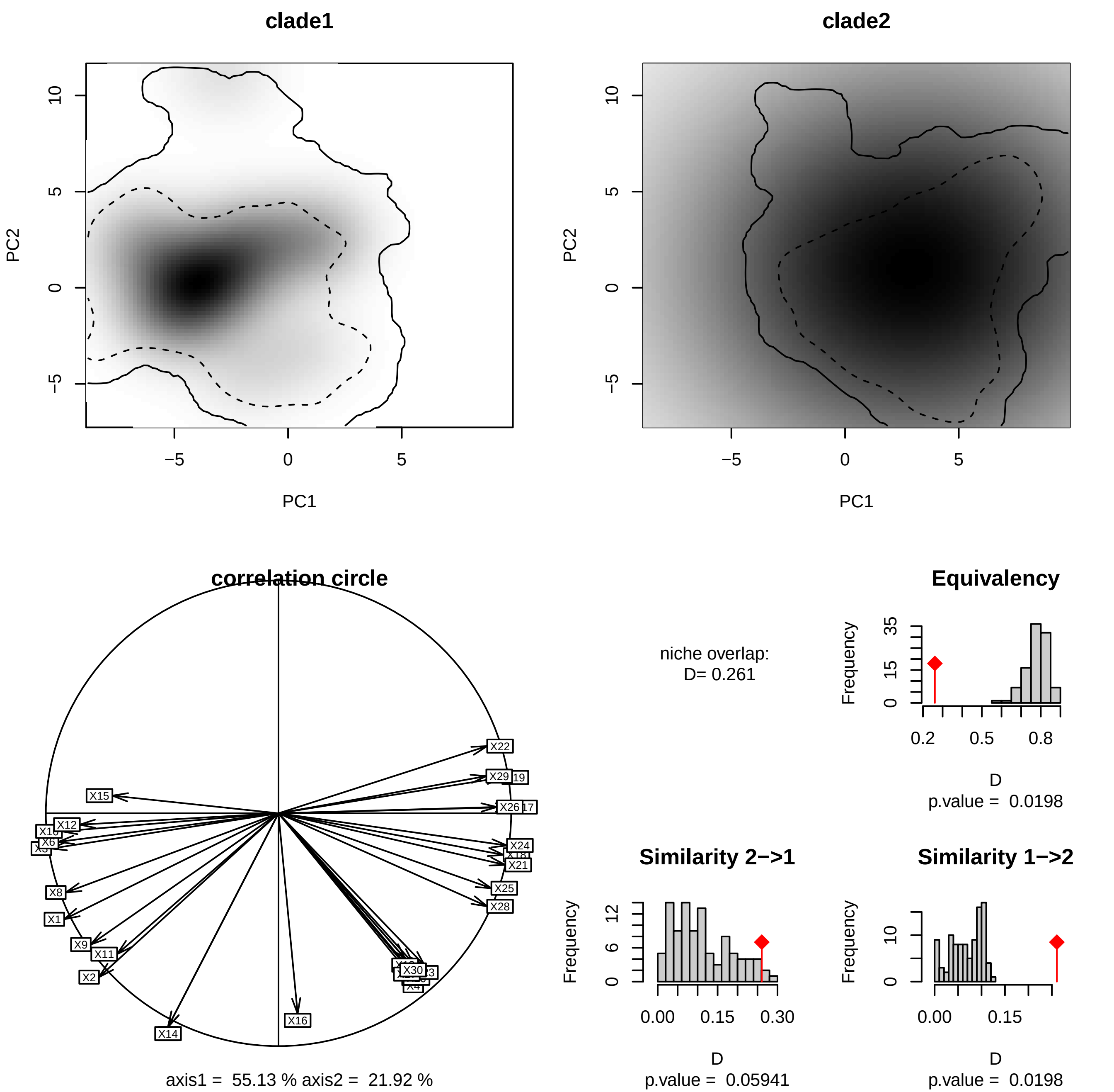
clade1
clade2
PC1
PC2
−5
0
5
10
correlation circle
X22
X29
X15
X26
X12
X6
X24
X21
X25
X28
X8
X1
X9
X11
X2
X30
X14
X16
axis1 = 55.13 % axis2 = 21.92 %
niche overlap:
D= 0.261
Equivalency
Frequency
D
p.value = 0.0198
Similarity 2−>1
p.value = 0.05941
Similarity 1−>2
p.value = 0.0198

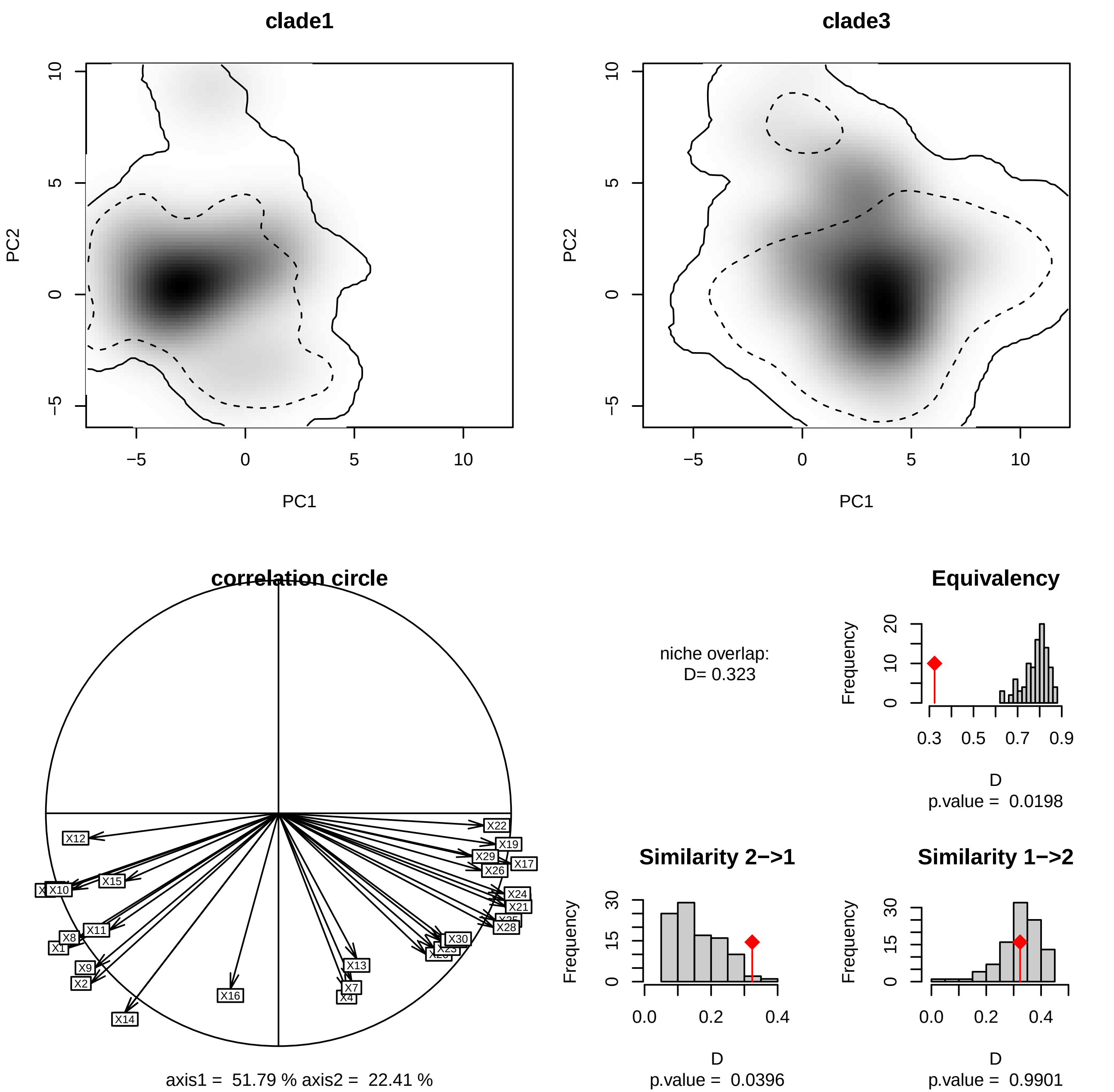
clade1
clade3
PC2
PC1
10
5
0
−5
−5
0
5
10
correlation circle
X22
X19
X29
X17
X26
X24
X21
X28
X30
X13
X7
X4
X16
X14
X12
X15
X10
X11
X8
X1
X9
X2
axis1 = 51.79 % axis2 = 22.41 %
niche overlap:
D= 0.323
Equivalency
Frequency
0
10
20
0.3
0.5
0.7
0.9
D
p.value = 0.0198
Similarity 2−>1
Frequency
0
15
30
0.0
0.2
0.4
D
p.value = 0.0396
Similarity 1−>2
Frequency
0
15
30
0.0
0.2
0.4
D
p.value = 0.9901

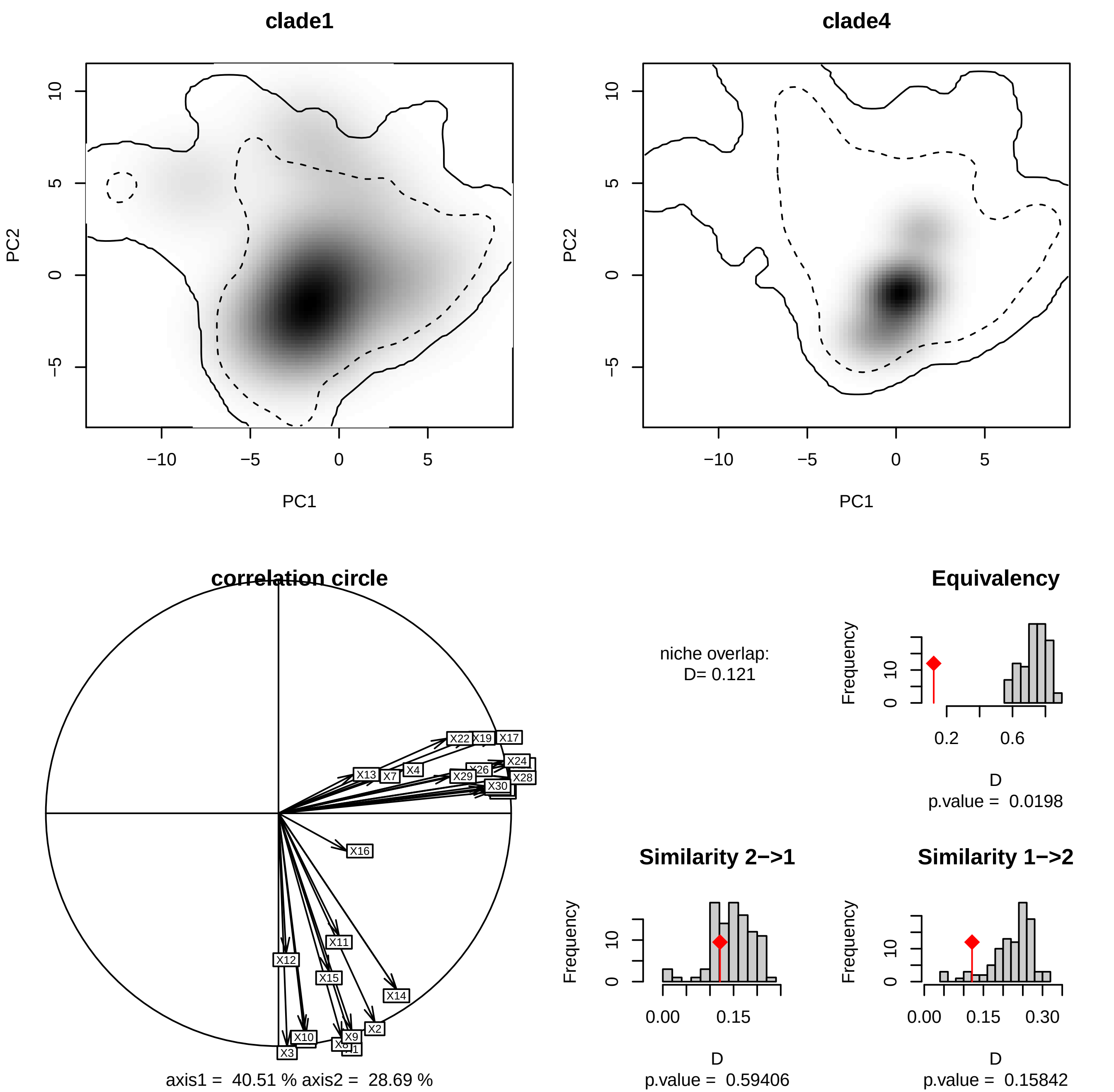

clade1
clade4
PC1
PC2
10
5
0
−5
−10
correlation circle
X22
X19
X17
X24
X13
X7
X4
X26
X29
X28
X30
X16
X11
X12
X15
X14
X2
X9
X10
X3
axis1 = 40.51 % axis2 = 28.69 %
niche overlap:
D= 0.121
Equivalency
Frequency
0.2
0.6
D
p.value = 0.0198
Similarity 2−>1
0.00
0.15
p.value = 0.59406
Similarity 1−>2
0.30
p.value = 0.15842

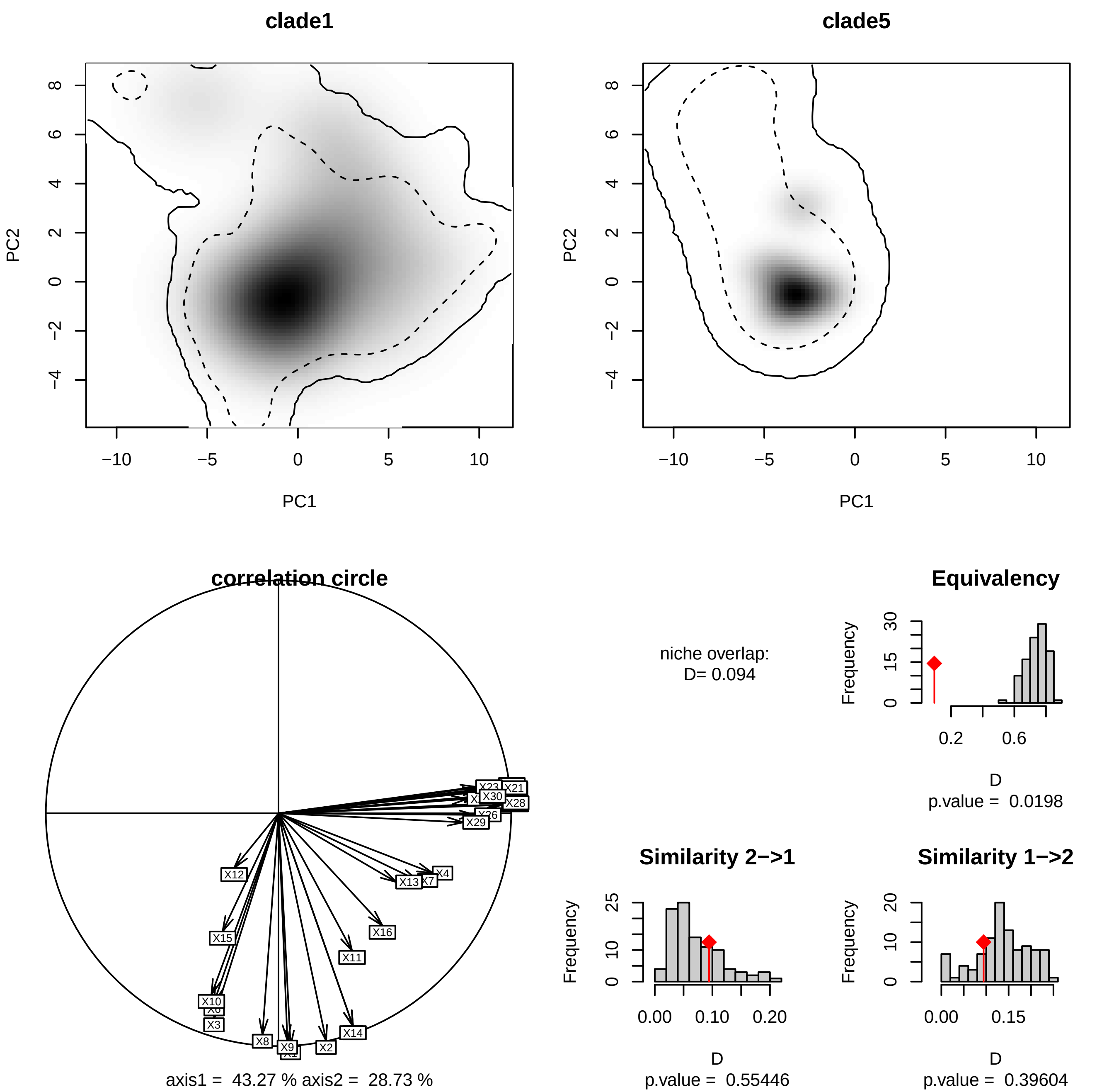

clade1
clade5
PC1
PC2
correlation circle
X21
X30
X28
X29
X12
X4
X7
X13
X15
X16
X11
X10
X3
X8
X9
X2
X14
axis1 = 43.27 % axis2 = 28.73 %
niche overlap:
D= 0.094
Equivalency
Frequency
D
p.value = 0.0198
Similarity 2−>1
p.value = 0.55446
Similarity 1−>2
p.value = 0.39604

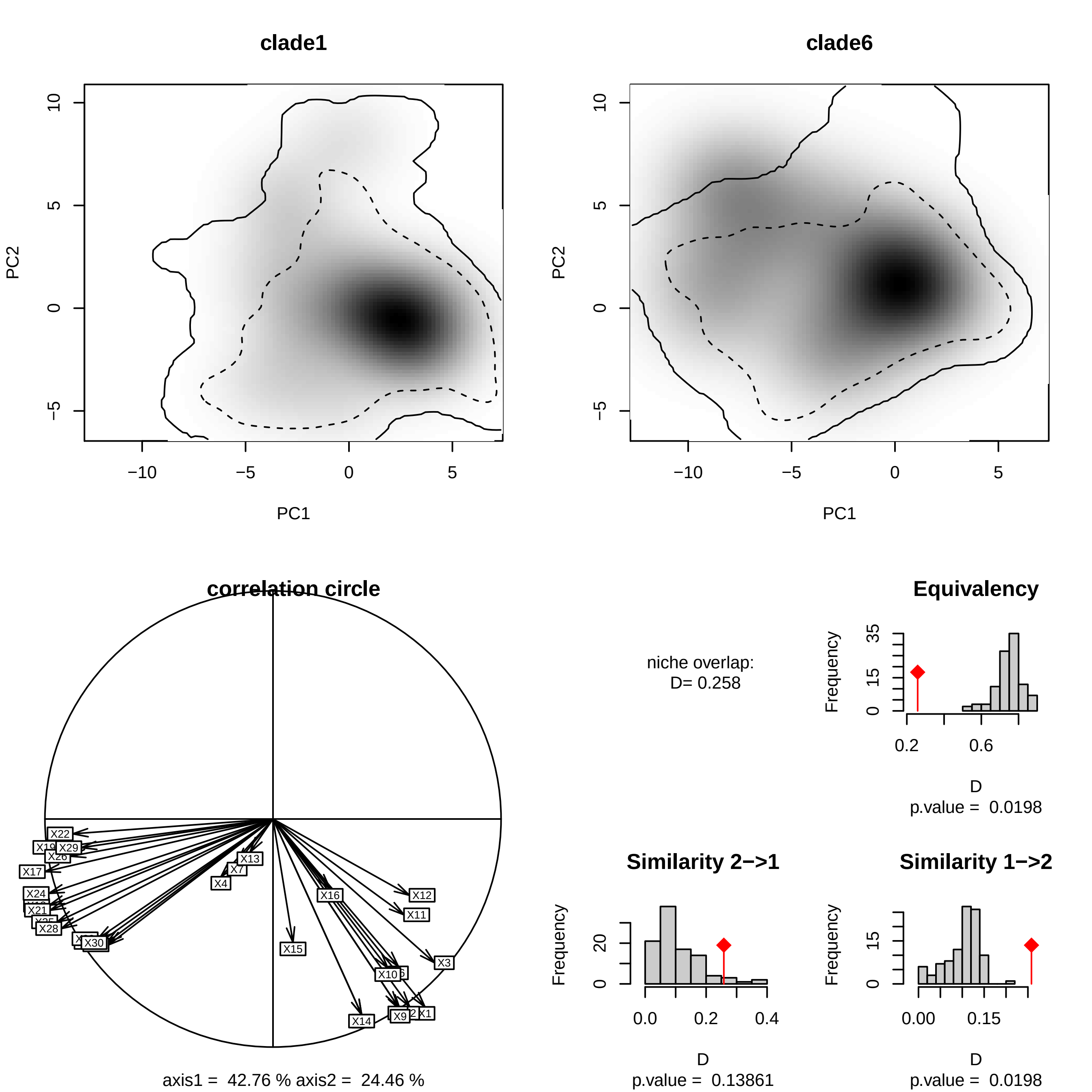
clade1
clade6
PC1
PC2
correlation circle
axis1 = 42.76 % axis2 = 24.46 %
X22
X29
X17
X24
X21
X28
X30
X13
X7
X4
X16
X12
X11
X15
X3
X10
X14
X9
X1
niche overlap:
D= 0.258
Equivalency
Frequency
D
p.value = 0.0198
Similarity 2−>1
Frequency
D
p.value = 0.13861
Similarity 1−>2
Frequency
D
p.value = 0.0198

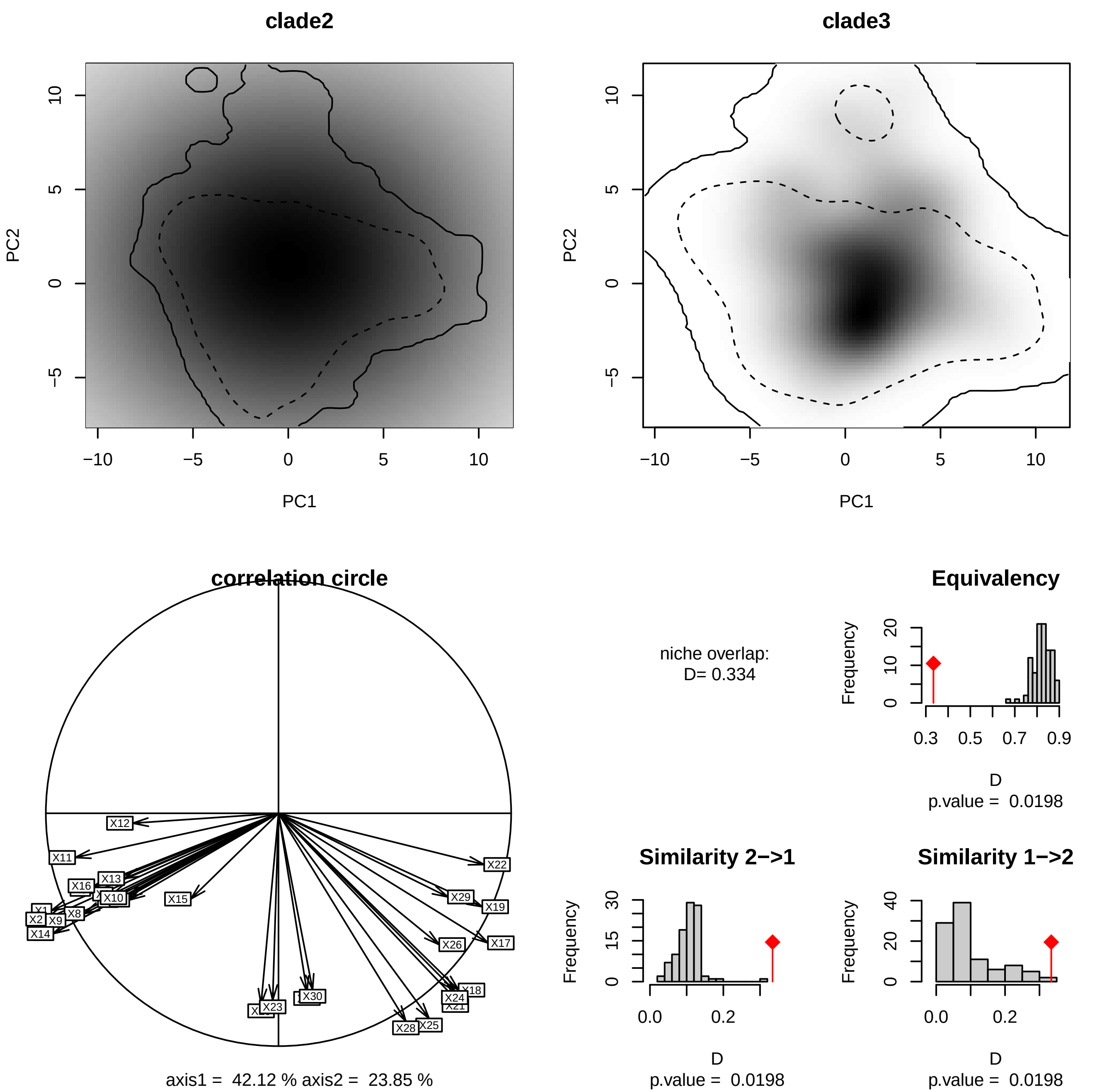
clade2
clade3
PC1
PC2
−10
−5
0
5
10
correlation circle
X12
X11
X13
X16
X10
X15
X1
X2
X9
X8
X14
X22
X29
X19
X17
X26
X18
X24
X21
X30
X23
X28
X25
axis1 = 42.12 % axis2 = 23.85 %
niche overlap:
D= 0.334
Equivalency
Frequency
D
p.value = 0.0198
Similarity 2−>1
Frequency
D
p.value = 0.0198
Similarity 1−>2
Frequency
D
p.value = 0.0198

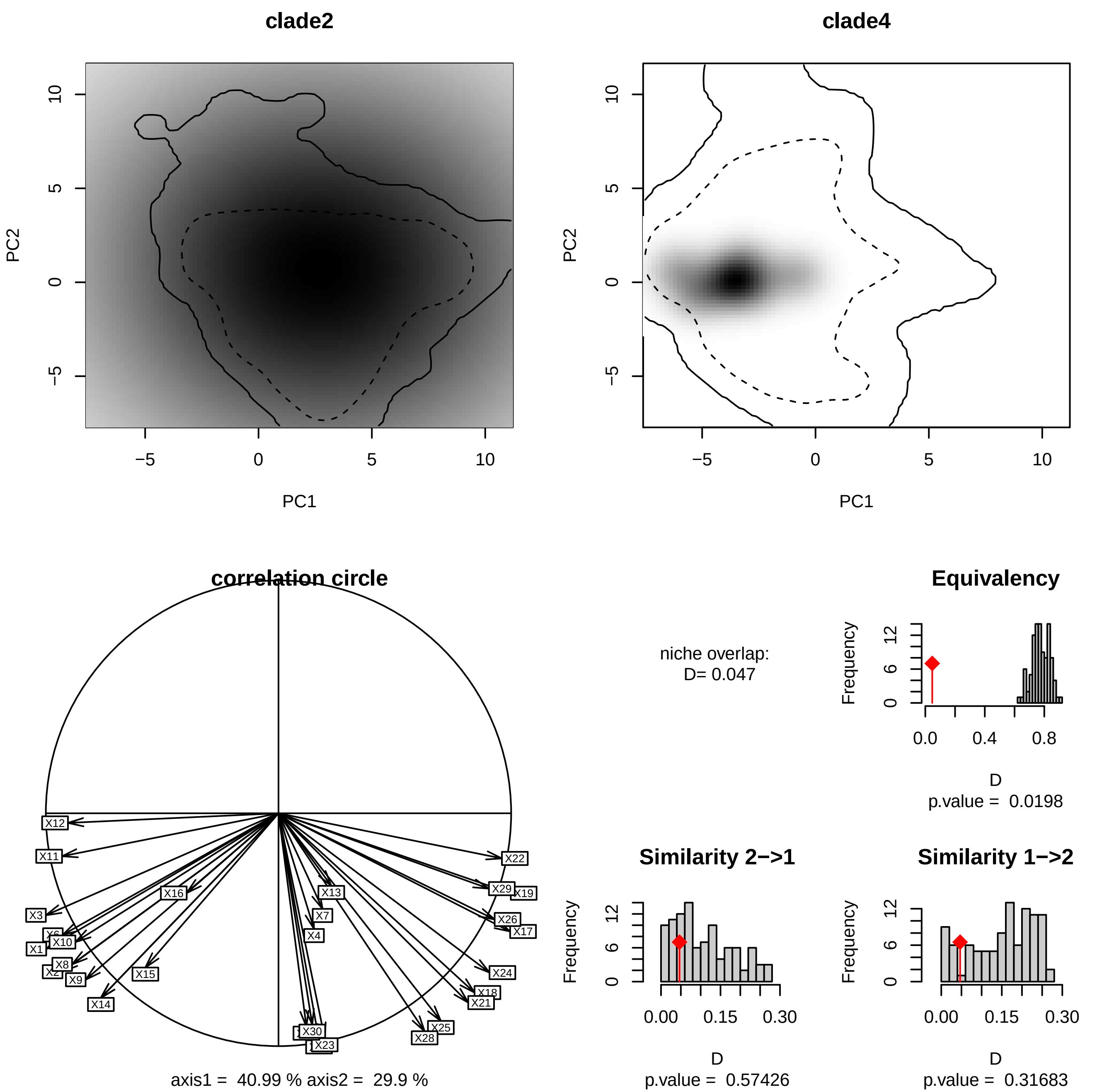
clade2
clade4
PC1
PC2
correlation circle
axis1 = 40.99 % axis2 = 29.9 %
niche overlap:
D= 0.047
Equivalency
Frequency
D
p.value = 0.0198
Similarity 2−>1
p.value = 0.57426
Similarity 1−>2
p.value = 0.31683

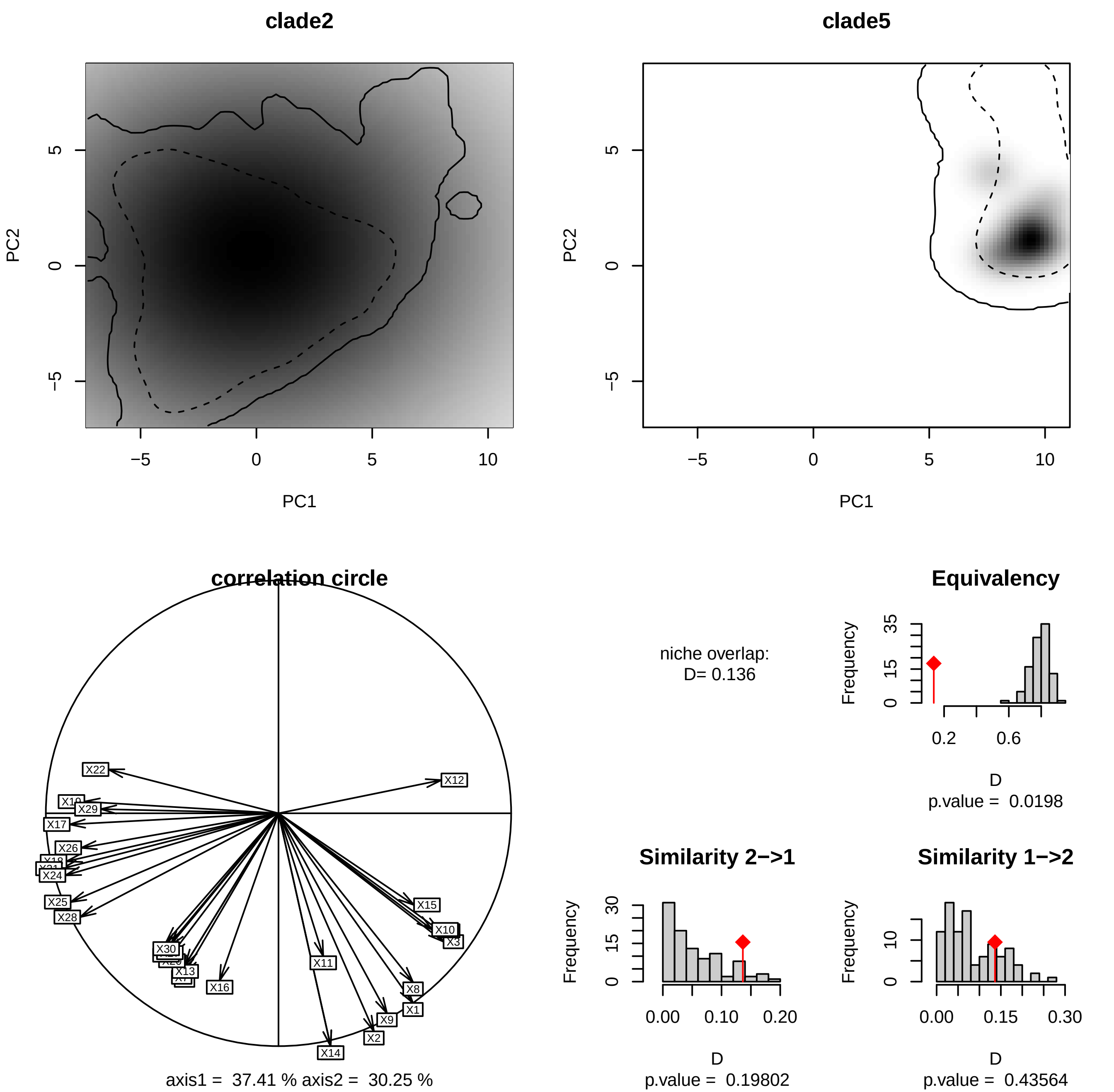
clade2
PC2
PC1
5
0
−5
10
clade5
correlation circle
X22
X12
X29
X17
X26
X24
X25
X28
X15
X10
X3
X30
X13
X16
X11
X8
X1
X9
X2
X14
axis1 = 37.41 % axis2 = 30.25 %
niche overlap:
D= 0.136
Equivalency
Frequency
35
15
0.2
0.6
D
p.value = 0.0198
Similarity 2−>1
30
0.00
0.10
0.20
p.value = 0.19802
Similarity 1−>2
0.15
0.30
p.value = 0.43564

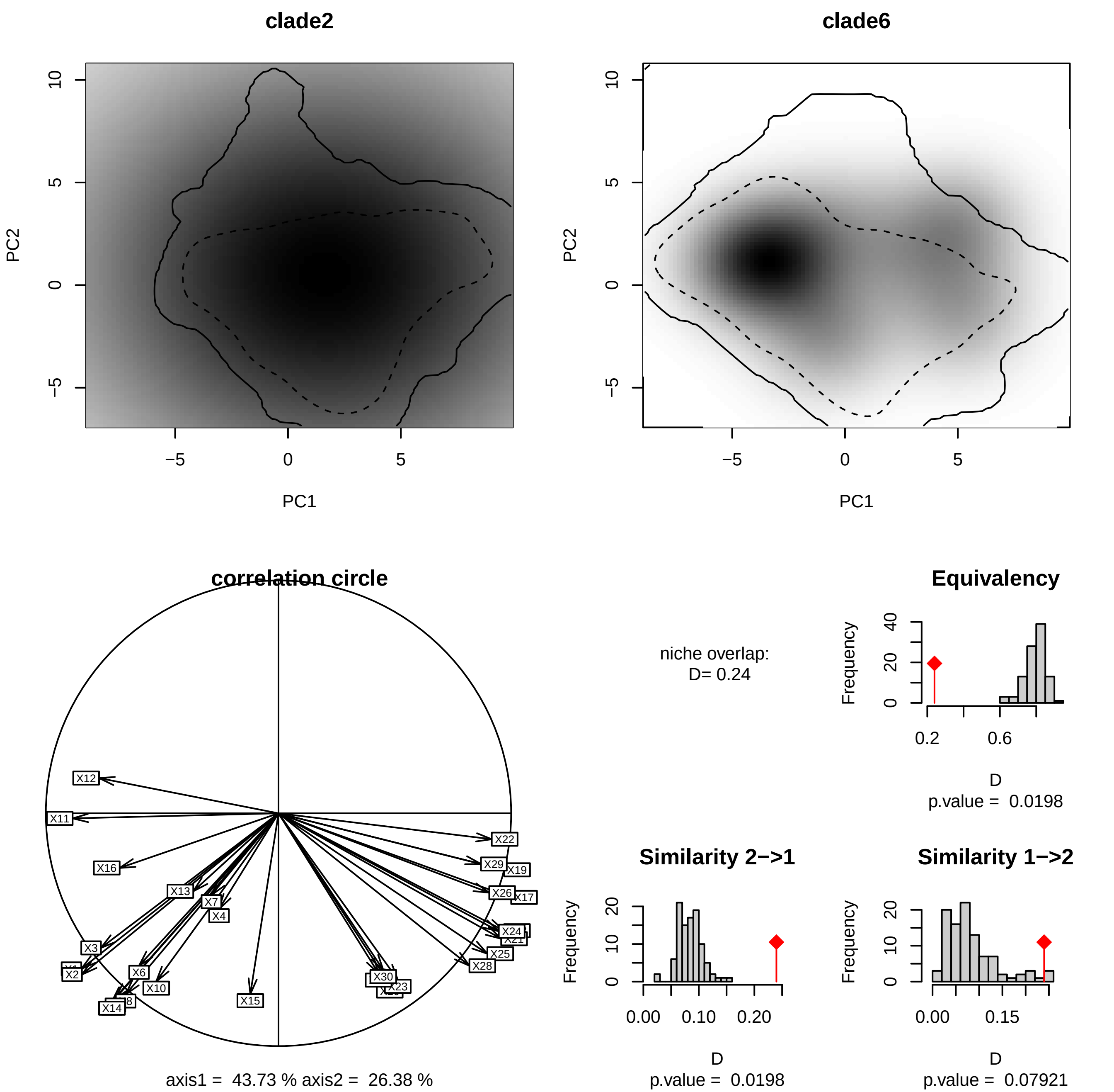
clade2
clade6
PC1
PC2
correlation circle
X12
X11
X16
X13
X7
X4
X3
X2
X6
X10
X14
X15
X30
X23
X22
X29
X19
X26
X17
X24
X25
X28
axis1 = 43.73 % axis2 = 26.38 %
niche overlap:
D= 0.24
Equivalency
Frequency
D
p.value = 0.0198
Similarity 2−>1
Frequency
D
p.value = 0.0198
Similarity 1−>2
Frequency
D
p.value = 0.07921

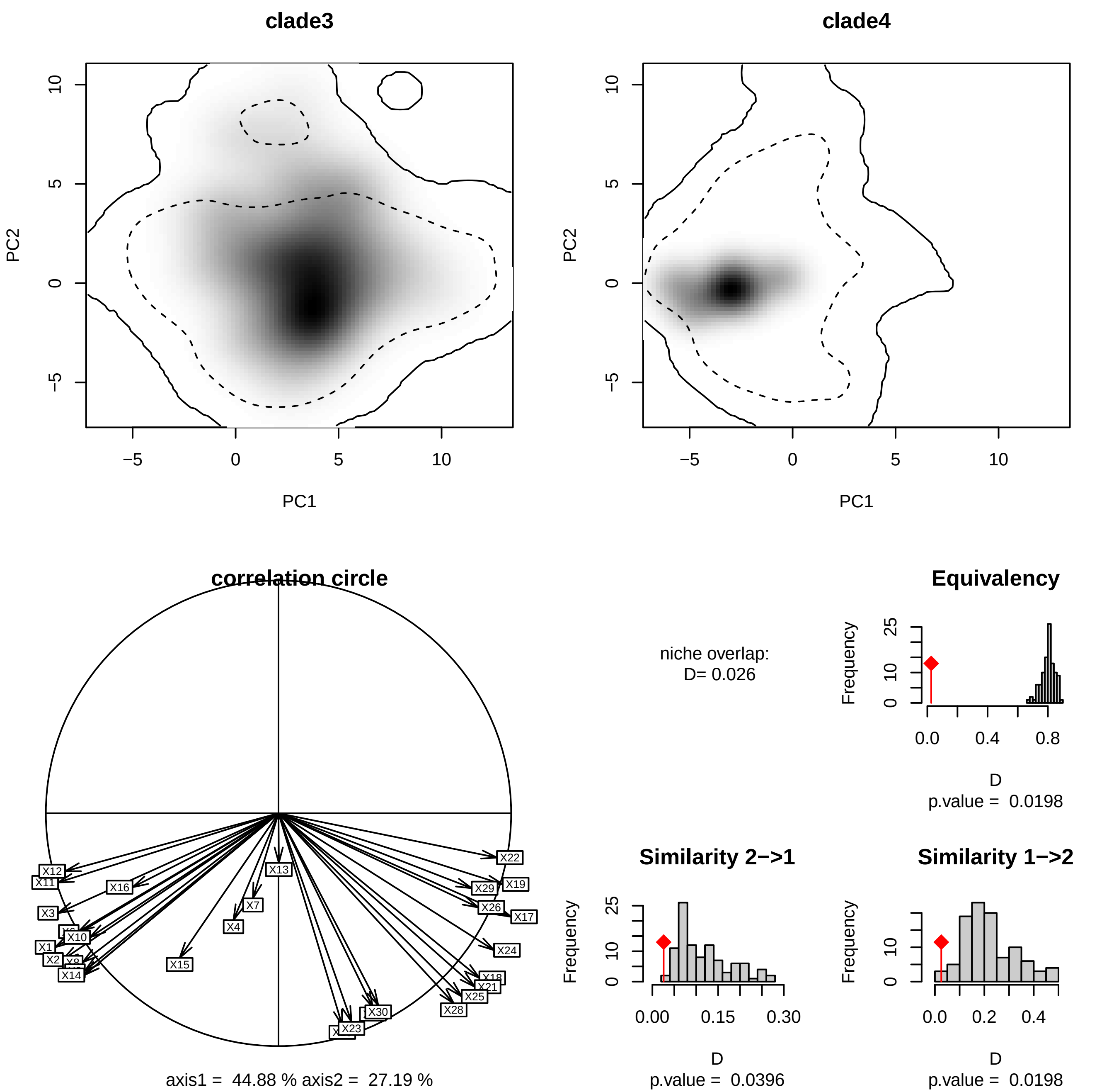
clade3
clade4
PC1
PC2
−5
0
5
10
correlation circle
X22
X12
X11
X16
X13
X19
X29
X26
X17
X3
X7
X4
X10
X24
X1
X2
X14
X15
X18
X21
X25
X28
X30
X23
axis1 = 44.88 % axis2 = 27.19 %
niche overlap:
D= 0.026
Equivalency
Frequency
D
p.value = 0.0198
Similarity 2−>1
Frequency
D
p.value = 0.0396
Similarity 1−>2
Frequency
D
p.value = 0.0198

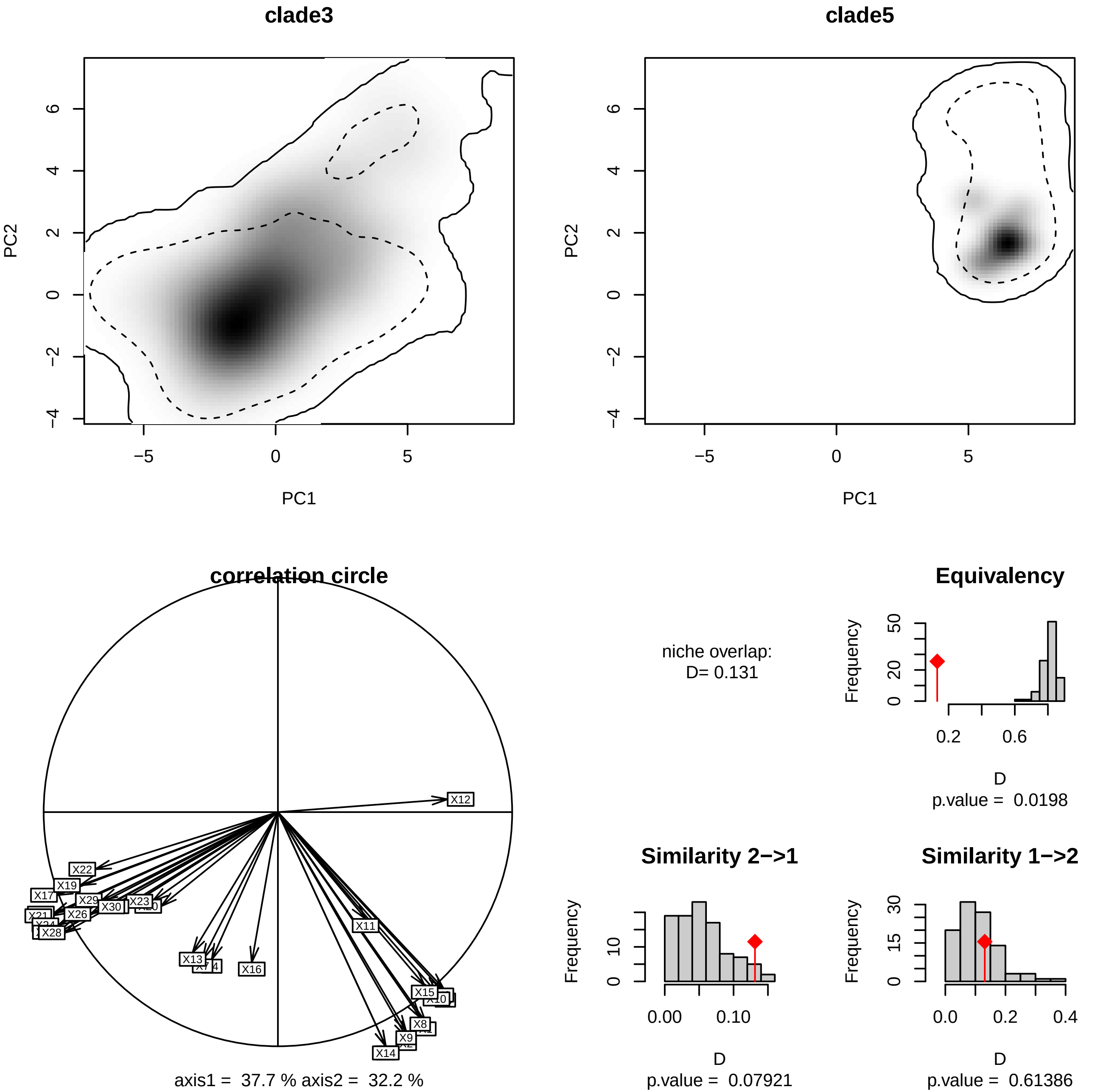
clade3
PC2
PC1
clade5
PC2
PC1
correlation circle
X12
X22
X19
X17
X29
X30
X23
X21
X26
X28
X11
X13
X16
X15
X8
X9
X14
axis1 = 37.7 % axis2 = 32.2 %
niche overlap:
D= 0.131
Equivalency
Frequency
D
p.value = 0.0198
Similarity 2−>1
Frequency
D
p.value = 0.07921
Similarity 1−>2
Frequency
D
p.value = 0.61386

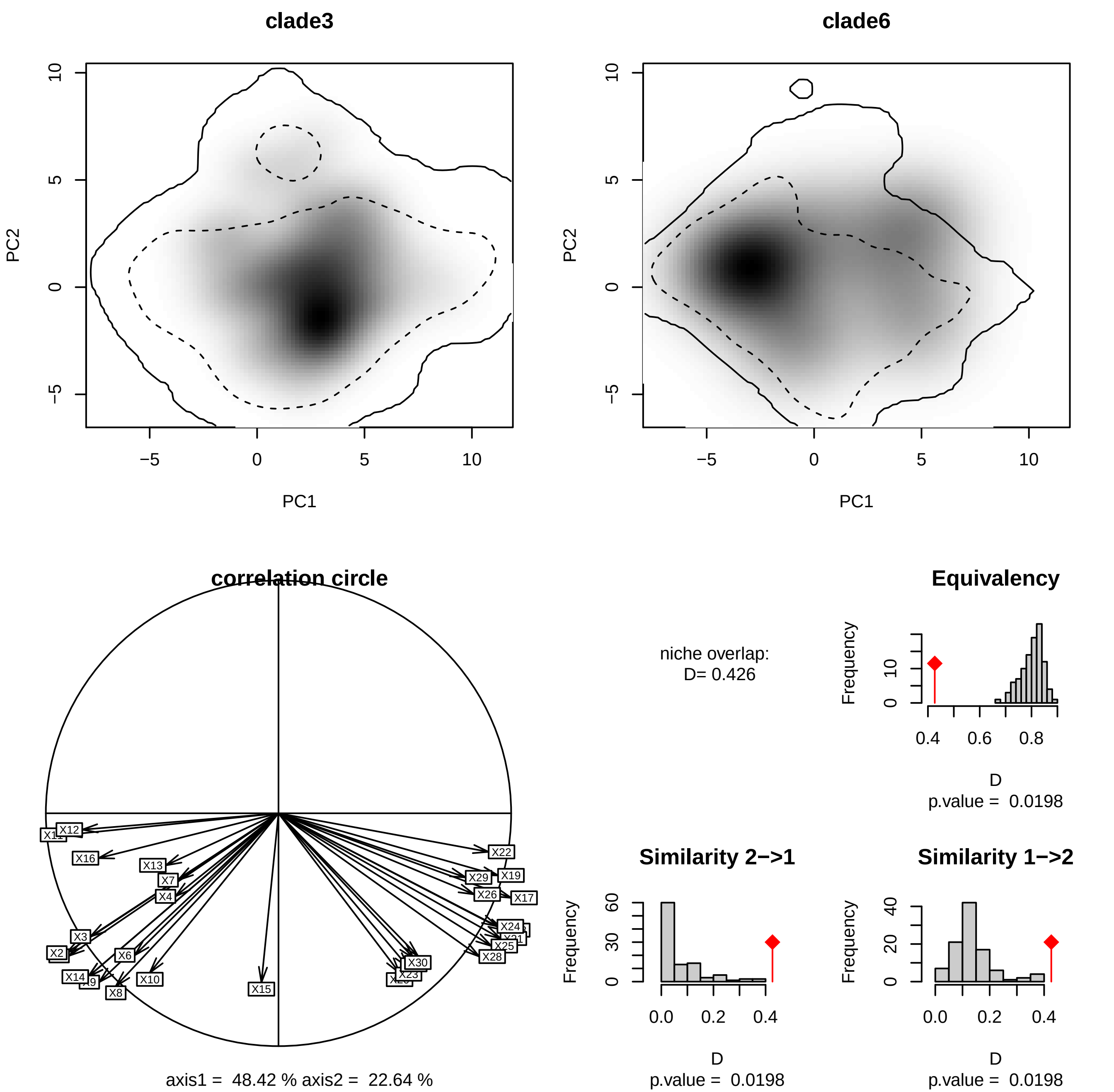
clade3
clade6
PC1
PC2
−5
0
5
10
correlation circle
X12
X16
X13
X7
X4
X3
X2
X6
X14
X8
X10
X15
X22
X29
X19
X26
X17
X24
X25
X28
X30
X23
axis1 = 48.42 % axis2 = 22.64 %
niche overlap:
D= 0.426
Equivalency
Frequency
0.4
0.6
0.8
D
p.value = 0.0198
Similarity 2−>1
Frequency
0.0
0.2
0.4
D
p.value = 0.0198
Similarity 1−>2
Frequency
0.0
0.2
0.4
D
p.value = 0.0198

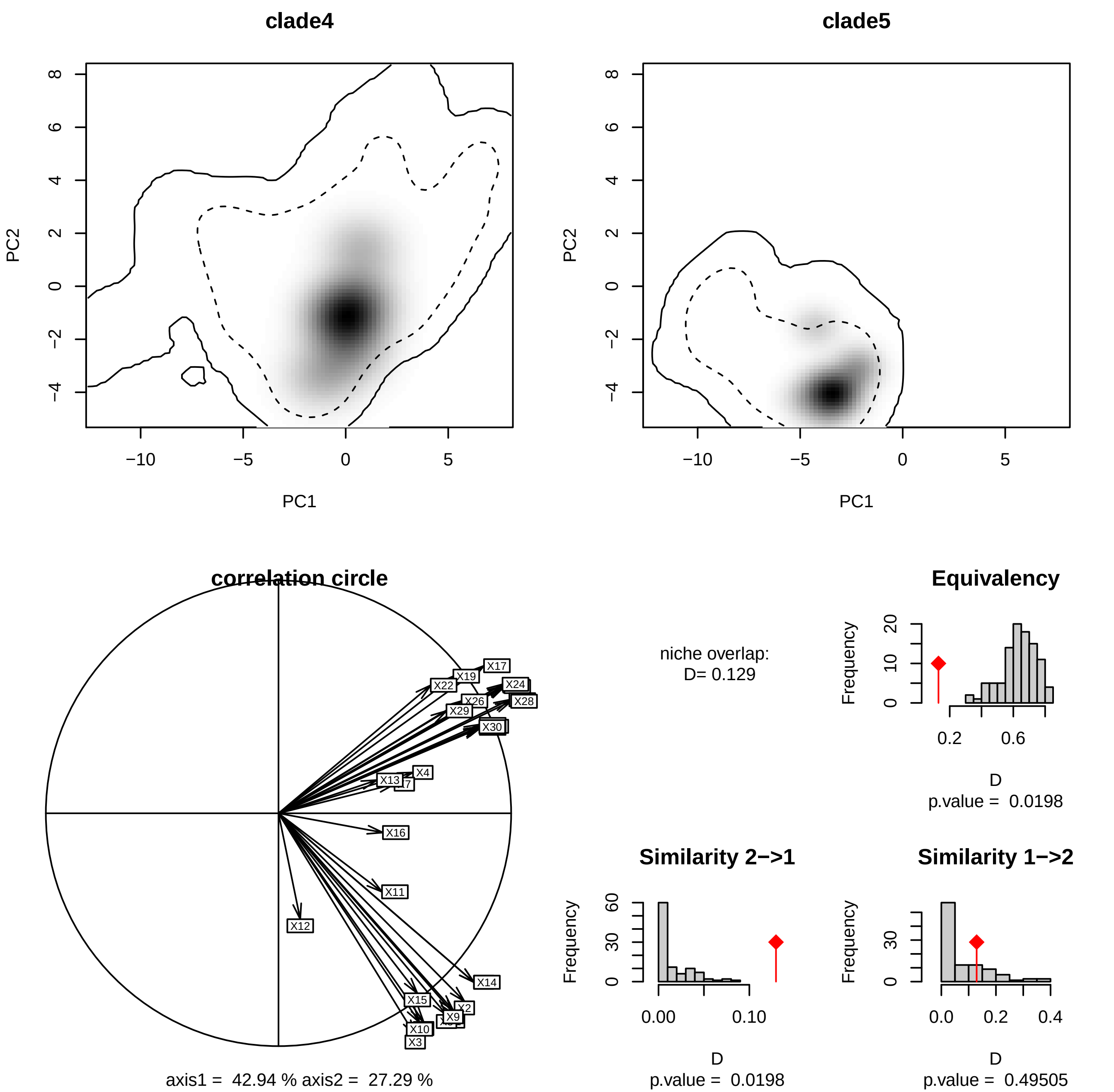
clade4
clade5
PC1
PC2
correlation circle
X17
X19
X22
X24
X26
X28
X29
X30
X4
X13
X16
X11
X12
X14
X15
X2
X9
X10
X3
axis1 = 42.94 % axis2 = 27.29 %
niche overlap:
D= 0.129
Equivalency
Frequency
D
p.value = 0.0198
Similarity 2−>1
Frequency
D
p.value = 0.0198
Similarity 1−>2
Frequency
D
p.value = 0.49505

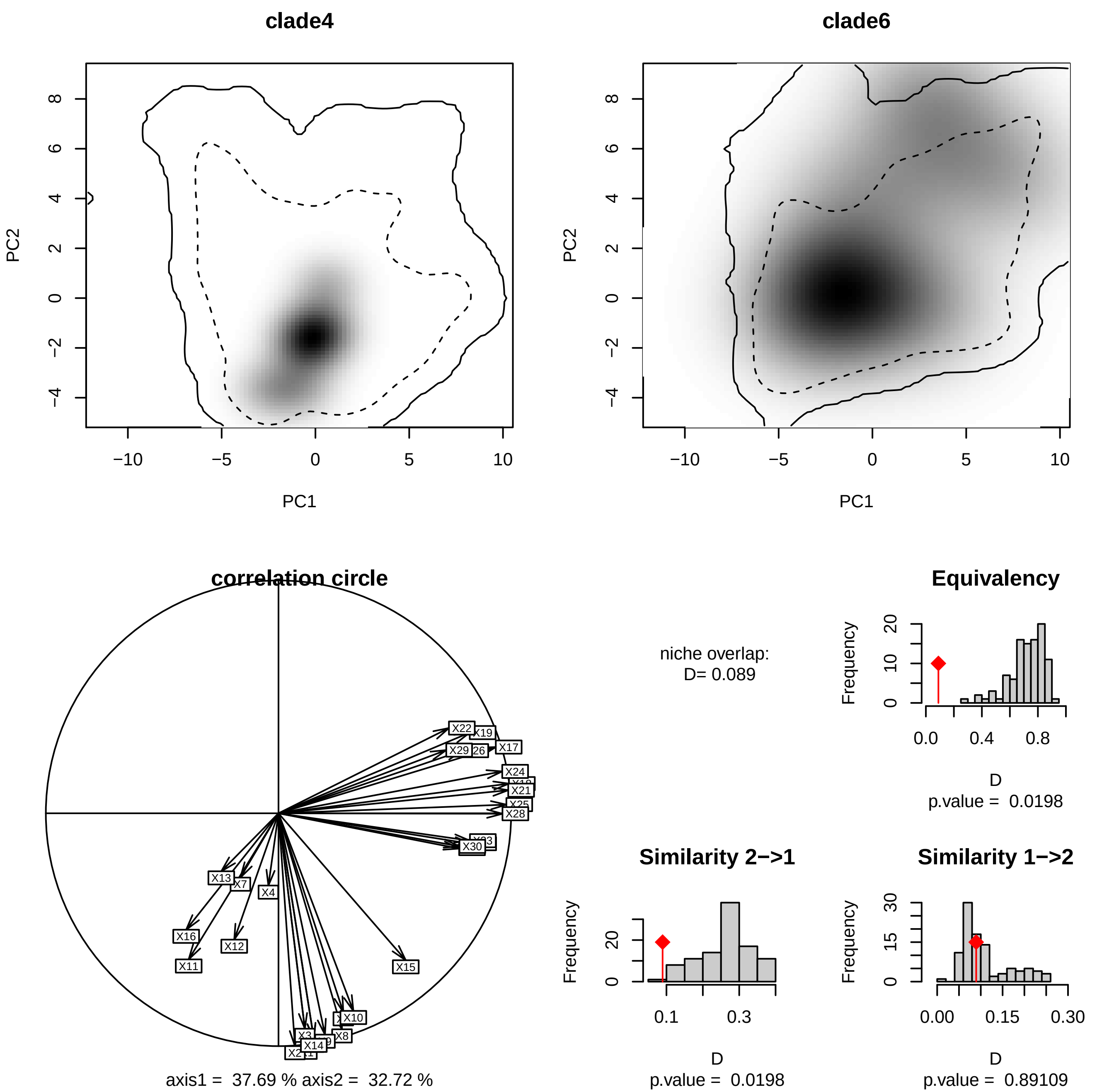
clade4
clade6
PC1
PC2
correlation circle
axis1 = 37.69 % axis2 = 32.72 %
X22
X19
X29
X17
X24
X21
X28
X30
X13
X7
X4
X16
X12
X11
X15
X10
X8
X3
X14
X2
niche overlap:
D= 0.089
Equivalency
Frequency
D
p.value = 0.0198
Similarity 2−>1
Frequency
D
p.value = 0.0198
Similarity 1−>2
Frequency
D
p.value = 0.89109

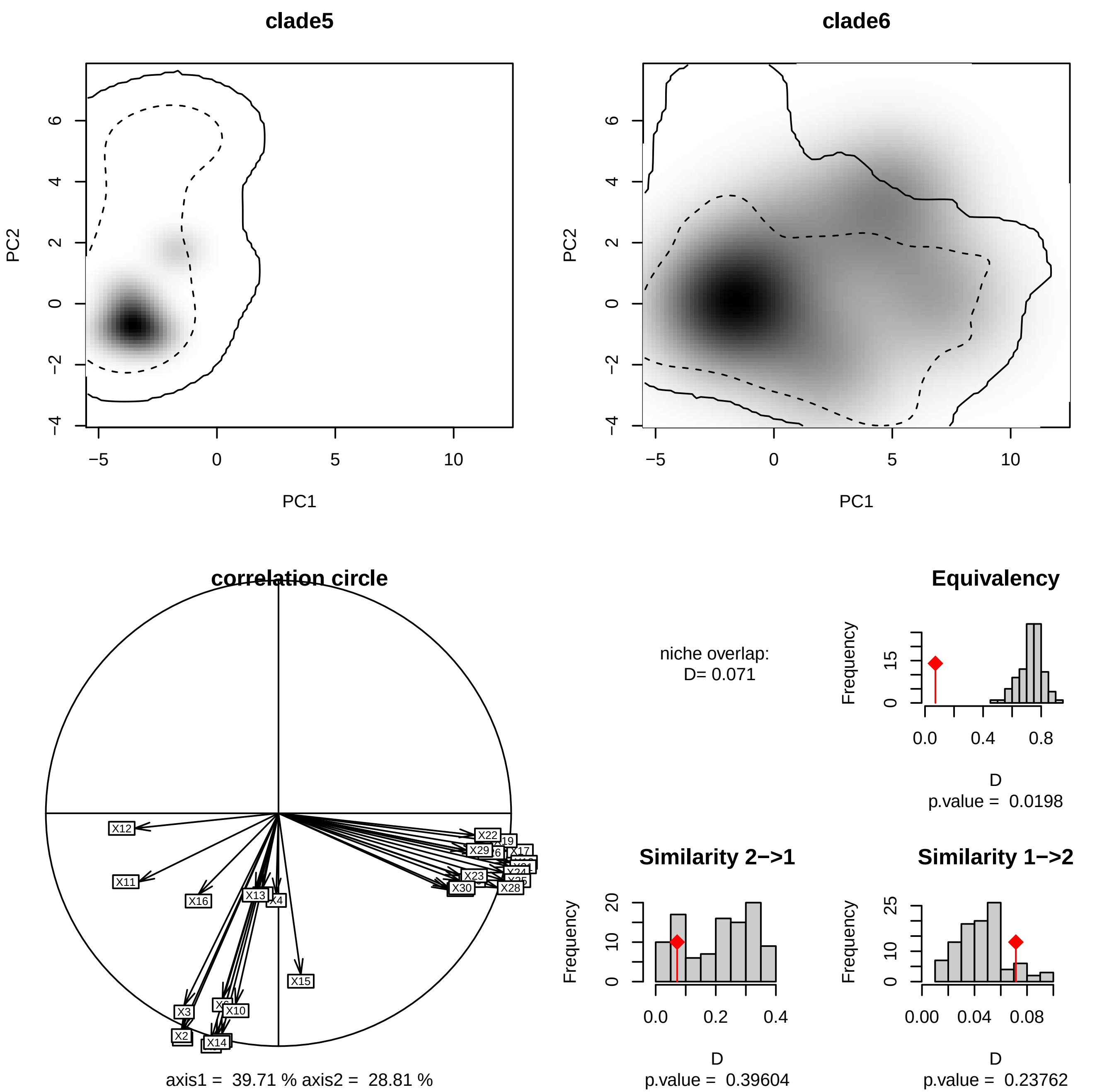
clade5
clade6
PC2
PC1
−5
0
5
10
−4
−2
2
4
6
correlation circle
X12
X11
X16
X13
X4
X15
X3
X2
X10
X14
X22
X29
X19
X17
X23
X30
X28
X24
X25
axis1 = 39.71 % axis2 = 28.81 %
niche overlap:
D= 0.071
Equivalency
Frequency
D
0.0
0.4
0.8
15
p.value = 0.0198
Similarity 2−>1
0.2
20
p.value = 0.39604
Similarity 1−>2
0.00
0.04
0.08
25
p.value = 0.23762
