# Supplementary material for: A Combination of Divergence and Conservatism in the Niche Evolution of the Moorish Gecko, Tarentola mauritanica (Gekkota: Phyllodactylidae)
Source: PLoS One. 2015 May 22;10(5):e0127980. doi: 10.1371/journal.pone.0127980 (PMC4441378; doi:10.1371/journal.pone.0127980)
Supplement: S1 Table — The results of the Principal Components analysis (PCA) include the component loadings and the correlation scores (in parenthesis) calculated using Pearson’s r correlation, for the first four principal components (PC). In both PC1 and PC2 the numbers in bold correspond to the variables that contributed more to each axis (absolute Pearson’s r correlation, |r| ≥ 0.8). The workflow to create the 30 variables was as following: the starting point were 5 sets of variables, each with 12 months = 60 single variables representing monthly means (12 x NLST; 12 x DLST; 12 x MIR; 12 x NDVI; 12 x EVI). For each of the set of 12 monthly variables of MIR, NDVI and EVI the following 7 temporal transformations were computed (= 21 new variables): BIO1 = Annual Mean; BIO4 = Seasonality; BIO5 = Max of Month with highest scores; BIO6 = Min of Month with lowest scores; BIO7 = Annual Range; BIO10 = Mean of Quarter with highest scores; BIO11 = Mean of the Quarter with lowest scores. Based on monthly variables of NLST and DLST the following 9 temporal transformations were computed (= 9 new variables): BIO1 = Annual Mean; BIO2 = Mean Diurnal Range; BIO3 = Isothermality; BIO4 = Seasonality; BIO5 = Max of Month with highest scores; BIO6 = Min of Month with lowest scores; BIO7 = Annual Range; BIO10 = Mean of Quarter with highest scores; BIO11 = Mean of the Quarter with lowest scores. These 30 new variables were subject to a PCA. (DOC) [file pone.0127980.s002.doc]

**S1 Table**. **Remote sensing variables and corresponding abbreviations used in this study**. The results of the Principal Components analysis (PCA) include the component loadings and the correlation scores (in parenthesis) calculated using Pearson’s r correlation, for the first four principal components (PC). In both PC1 and PC2 the numbers in bold correspond to the variables that contributed more to each axis (absolute Pearson’s r correlation, |r| ≥ 0.8). The workflow to create the 30 variables was as following: the starting point were 5 sets of variables, each with 12 months = 60 single variables representing monthly means (12 x NLST; 12 x DLST; 12 x MIR; 12 x NDVI; 12 x EVI). For each of the set of 12 monthly variables of MIR, NDVI and EVI the following 7 temporal transformations were computed (= 21 new variables): BIO1 = Annual Mean; BIO4 = Seasonality; BIO5 = Max of Month with highest scores; BIO6 = Min of Month with lowest scores; BIO7 = Annual Range; BIO10 = Mean of Quarter with highest scores; BIO11 = Mean of the Quarter with lowest scores. Based on monthly variables of NLST and DLST the following 9 temporal transformations were computed (= 9 new variables): BIO1 = Annual Mean; BIO2 = Mean Diurnal Range; BIO3 = Isothermality; BIO4 = Seasonality; BIO5 = Max of Month with highest scores; BIO6 = Min of Month with lowest scores; BIO7 = Annual Range; BIO10 = Mean of Quarter with highest scores; BIO11 = Mean of the Quarter with lowest scores. These 30 new variables were subject to a PCA.

| **Variable** | **Abbreviation** | **Meaning Sat Variable** | **Temporal Transformation** | **PC1** | **PC2** | **PC3** | **PC4** |
| --- | --- | --- | --- | --- | --- | --- | --- |
| **X1** | ED1503_bio1 | MODIS V4 Band 03 Synoptic Months: Middle Infra-Red | BIO1 = Annual Mean | **-0.96 (0.93)** | 0.05 (0) | -0.18 (0.03) | 0.18 (0) |
| **X2** | ED1503_bio10 | MODIS V4 Band 03 Synoptic Months: Middle Infra-Red | BIO10 = Mean of Quarter with highest scores | -**0.93 (0.87)** | -0.06 (0) | -0.33 (0.08) | 0.14 (0) |
| **X3** | ED1503_bio11 | MODIS V4 Band 03 Synoptic Months: Middle Infra-Red | BIO11 = Mean of the Quarter with lowest scores | **-0.96 (0.92)** | 0.2 (0.05) | -0.04 (0) | 0.19 (0.01) |
| **X4** | ED1503_bio4 | MODIS V4 Band 03 Synoptic Months: Middle Infra-Red | BIO4 = Seasonality | 0.54 (0.28) | -0.58 (0.42) | -0.51 (0.24) | -0.21 (0.04) |
| **X5** | ED1503_bio5 | MODIS V4 Band 03 Synoptic Months: Middle Infra-Red | BIO5 = Max of Month with highest scores | **-0.89 (0.8)** | -0.11 (0.02) | -0.41 (0.13) | 0.12 (0) |
| **X6** | ED1503_bio6 | MODIS V4 Band 03 Synoptic Months: Middle Infra-Red | BIO6 = Min of Month with lowest scores | **-0.94 (0.89)** | 0.24 (0.06) | 0.01 (0) | 0.21 (0.01) |
| **X7** | ED1503_bio7 | MODIS V4 Band 03 Synoptic Months: Middle Infra-Red | BIO7 = Annual Range | 0.55 (0.29) | -0.56 (0.39) | -0.52 (0.24) | -0.23 (0.06) |
| **X8** | ED150708_bio1 | MODIS V4 Band 07 + 08 Synoptic Months: Day- + Night-time Land Surface Temperature | BIO1 = Annual Mean | **-0.95 (0.89)** | 0.11 (0.01) | -0.18 (0.03) | 0.16 (0) |
| **X9** | ED150708_bio10 | MODIS V4 Band 07 + 08 Synoptic Months: Day- + Night-time Land Surface Temperature | BIO10 = Mean of Quarter with highest scores | **-0.93 (0.87)** | 0 (0) | -0.28 (0.07) | 0.14 (0) |
| **X10** | ED150708_bio11 | MODIS V4 Band 07 + 08 Synoptic Months: Day- + Night-time Land Surface Temperature | BIO11 = Mean of the Quarter with lowest scores | **-0.94 (0.87)** | 0.27 (0.07) | -0.09 (0.01) | 0.15 (0.01) |
| **X11** | ED150708_bio2 | MODIS V4 Band 07 + 08 Synoptic Months: Day- + Night-time Land Surface Temperature | BIO2 = Mean Diurnal Range | **-0.91 (0.85)** | -0.09 (0) | -0.17 (0.01) | 0.2 (0) |
| **X12** | ED150708_bio3 | MODIS V4 Band 07 + 08 Synoptic Months: Day- + Night-time Land Surface Temperature | BIO3 = Isothermality | **-0.89 (0.78)** | 0.17 (0.06) | -0.02 (0) | 0.21 (0.01) |
| **X13** | ED150708_bio4 | MODIS V4 Band 07 + 08 Synoptic Months: Day- + Night-time Land Surface Temperature | BIO4 = Seasonality | 0.36 (0.09) | -0.76 (0.65) | -0.47 (0.19) | -0.11 (0.05) |
| **X14** | ED150708_bio5 | MODIS V4 Band 07 + 08 Synoptic Months: Day- + Night-time Land Surface Temperature | BIO5 = Max of Month with highest scores | **-0.89 (0.8)** | -0.11 (0.02) | -0.41 (0.13) | 0.12 (0) |
| **X15** | ED150708_bio6 | MODIS V4 Band 07 + 08 Synoptic Months: Day- + Night-time Land Surface Temperature | BIO6 = Min of Month with lowest scores | -0.66 (0.46) | 0.42 (0.16) | -0.18 (0.05) | 0 (0.01) |
| **X16** | ED150708_bio7 | MODIS V4 Band 07 + 08 Synoptic Months: Day- + Night-time Land Surface Temperature | BIO7 = Annual Range | -0.69 (0.49) | -0.45 (0.21) | -0.41 (0.11) | 0.15 (0.02) |
| **X17** | ED1514_bio1 | MODIS V4 Band 14 Synoptic Months: Normalised Difference Vegetation Index | BIO1 = Annual Mean | **0.93 (0.88)** | 0.27 (0.06) | -0.18 (0.05) | 0.07 (0) |
| **X18** | ED1514_bio10 | MODIS V4 Band 14 Synoptic Months: Normalised Difference Vegetation Index | BIO10 = Mean of Quarter with highest scores | **0.95 (0.92)** | 0.17 (0.02) | -0.16 (0.05) | 0.14 (0.02) |
| **X19** | ED1514_bio11 | MODIS V4 Band 14 Synoptic Months: Normalised Difference Vegetation Index | BIO11 = Mean of the Quarter with lowest scores | 0.79 (0.67) | 0.49 (0.19) | -0.32 (0.13) | -0.05 (0.01) |
| **X20** | ED1514_bio4 | MODIS V4 Band 14 Synoptic Months: Normalised Difference Vegetation Index | BIO4 = Seasonality | **0.81 (0.62)** | -0.37 (0.15) | 0.12 (0.01) | 0.36 (0.22) |
| **X21** | ED1514_bio5 | MODIS V4 Band 14 Synoptic Months: Normalised Difference Vegetation Index | BIO5 = Max of Month with highest scores | **0.97 (0.94)** | 0.1 (0.01) | -0.12 (0.03) | 0.17 (0.03) |
| **X22** | ED1514_bio6 | MODIS V4 Band 14 Synoptic Months: Normalised Difference Vegetation Index | BIO6 = Min of Month with lowest scores | 0.74 (0.59) | 0.55 (0.24) | -0.32 (0.12) | -0.08 (0.02) |
| **X23** | ED1514_bio7 | MODIS V4 Band 14 Synoptic Months: Normalised Difference Vegetation Index | BIO7 = Annual Range | **0.83 (0.66)** | -0.36 (0.14) | 0.11 (0) | 0.34 (0.21) |
| **X24** | ED1515_bio1 | MODIS V4 Band 15 Synoptic Months: Enhanced Vegetation Index | BIO1 = Annual Mean | **0.91 (0.84)** | 0.26 (0.06) | -0.22 (0.09) | 0.16 (0.01) |
| **X25** | ED1515_bio10 | MODIS V4 Band 15 Synoptic Months: Enhanced Vegetation Index | BIO10 = Mean of Quarter with highest scores | **0.94 (0.89)** | 0.15 (0.03) | -0.16 (0.06) | 0.23 (0.03) |
| **X26** | ED1515_bio11 | MODIS V4 Band 15 Synoptic Months: Enhanced Vegetation Index | BIO11 = Mean of the Quarter with lowest scores | 0.69 (0.56) | 0.54 (0.2) | -0.43 (0.2) | -0.03 (0.01) |
| **X27** | ED1515_bio4 | MODIS V4 Band 15 Synoptic Months: Enhanced Vegetation Index | BIO4 = Seasonality | **0.87 (0.77)** | -0.23 (0.04) | 0.1 (0) | 0.39 (0.2) |
| **X28** | ED1515_bio5 | MODIS V4 Band 15 Synoptic Months: Enhanced Vegetation Index | BIO5 = Max of Month with highest scores | **0.95 (0.9)** | 0.06 (0.01) | -0.11 (0.04) | 0.27 (0.06) |
| **X29** | ED1515_bio6 | MODIS V4 Band 15 Synoptic Months: Enhanced Vegetation Index | BIO6 = Min of Month with lowest scores | 0.62 (0.48) | 0.6 (0.26) | -0.44 (0.21) | -0.07 (0.03) |
| **X30** | ED1515_bio7 | MODIS V4 Band 15 Synoptic Months: Enhanced Vegetation Index | BIO7 = Annual Range | **0.88 (0.79)** | -0.23 (0.03) | 0.08 (0) | 0.38 (0.18) |
| **Eigenvalues** |  |  |  | 21.31 | 3.63 | 2.36 | 1.21 |
| **Explained Variance (%)** |  |  |  | 71.04 | 12.11 | 7.88 | 4.02 |
