# Supplementary material for: A Combination of Divergence and Conservatism in the Niche Evolution of the Moorish Gecko, Tarentola mauritanica (Gekkota: Phyllodactylidae)
Source: PLoS One. 2015 May 22;10(5):e0127980. doi: 10.1371/journal.pone.0127980 (PMC4441378; doi:10.1371/journal.pone.0127980)
Supplement: S2 Table — Individuals and their corresponding clade assignation and geographic coordinates. (DOC) [file pone.0127980.s003.doc]

**S2 Table.** **Specimens used in this study**. Individuals and their corresponding clade assignation and geographic coordinates.

| **Species** | **Specimen Code** | **Clade** | **Latitude** | **Longitud** |
| --- | --- | --- | --- | --- |
| *Tarentola angustimentalis* | DB169 | Clade 5 | 28.42503056 | -14.00516111 |
|  | DB1318 | Clade 5 | 29.04646333 | -13.56205667 |
|  | DB1326 | Clade 5 | 28.95179333 | -13.76787833 |
|  | DB1334 | Clade 5 | 29.04646333 | -13.56205667 |
|  | DB1340 | Clade 5 | 28.30018667 | -14.02458333 |
|  | DB1353 | Clade 5 | 29.19772167 | -13.48401 |
|  | DB1354 | Clade 5 | 28.51435333 | -14.00315167 |
|  | DB1357 | Clade 5 | 28.51435333 | -14.00315167 |
|  | DB1358 | Clade 5 | 28.95179333 | -13.76787833 |
|  | DB1365 | Clade 5 | 28.30018667 | -14.02458333 |
|  | DB1373 | Clade 5 | 28.67987167 | -13.93198667 |
|  | DB1374 | Clade 5 | 28.60748667 | -13.92562667 |
|  | DB1389 | Clade 5 | 28.60748667 | -13.92562667 |
|  | DB1402 | Clade 5 | 29.19772167 | -13.48401 |
|  | DB1469 | Clade 5 | 29.19772167 | -13.48401 |
|  | DB7140 | Clade 5 | 28.07621 | -14.30856 |
|  | DB11988 | Clade 5 | 29.04646333 | -13.56205667 |
|  | DB13277 | Clade 5 | 28.95179333 | -13.76787833 |
|  | DB13730 | Clade 5 | 29.06636 | -13.58725 |
|  | DB13731 | Clade 5 | 29.06636 | -13.58725 |
|  | DB16511 | Clade 5 | 28.60748667 | -13.92562667 |
| *Tarentola mauritanica* | DB151 | Clade 3 | 36.13964 | -5.838254 |
|  | DB152 | Clade 3 | 36.13964 | -5.838254 |
|  | DB153 | Clade 3 | 41.37447222 | 2.149841667 |
|  | DB154 | Clade 3 | 36.372613 | -6.167205 |
|  | DB155 | Clade 3 | 36.372613 | -6.167205 |
|  | DB156 | Clade 3 | 37.31593611 | -8.824763889 |
|  | DB157 | Clade 3 | 37.31593611 | -8.824763889 |
|  | DB159 | Clade 6 | 34.41581944 | -4.480836111 |
|  | DB160 | Clade 1 | 30.83 | -8.33 |
|  | DB161 | Clade 3 | 36.816667 | 9.266667 |
|  | DB162 | Clade 3 | 36.871492 | 8.746268 |
|  | DB163 | Clade 3 | 36.94521389 | 8.757544444 |
|  | DB164 | Clade 3 | 40.853845 | 14.241213 |
|  | DB165 | Clade 6 | 34.21920833 | -4.005988889 |
|  | DB166 | Clade 3 | 32.72639722 | -16.93413056 |
|  | DB173 | Clade 3 | 35.1868 | 24.95276111 |
|  | DB176 | Clade 3 | 38.40373889 | -6.419225 |
|  | DB177 | Clade 3 | 38.40373889 | -6.419225 |
|  | DB178 | Clade 3 | 38.40373889 | -6.419225 |
|  | DB179 | Clade 3 | 37.86122222 | -6.479033333 |
|  | DB181 | Clade 1 | 32.94926389 | -8.206080556 |
|  | DB182 | Clade 1 | 32.94926389 | -8.206080556 |
|  | DB183 | Clade 1 | 32.94926389 | -8.206080556 |
|  | DB184 | Clade 1 | 32.79001667 | -6.961177778 |
|  | DB185 | Clade 1 | 32.79001667 | -6.961177778 |
|  | DB186 | Clade 1 | 32.79001667 | -6.961177778 |
|  | DB187 | Clade 1 | 32.79001667 | -6.961177778 |
|  | DB188 | Clade 1 | 32.79001667 | -6.961177778 |
|  | DB189 | Clade 1 | 32.79001667 | -6.961177778 |
|  | DB190 | Clade 1 | 31.2445 | -7.98449 |
|  | DB191 | Clade 1 | 31.2445 | -7.98449 |
|  | DB192 | Clade 1 | 30.83610833 | -8.954413889 |
|  | DB193 | Clade 1 | 30.83610833 | -8.954413889 |
|  | DB195 | Clade 1 | 30.828272 | -9.132838 |
|  | DB197 | Clade 1 | 29.70666667 | -9.726777778 |
|  | DB198 | Clade 1 | 29.70666667 | -9.726777778 |
|  | DB199 | Clade 1 | 29.95 | -9.633333 |
|  | DB200 | Clade 1 | 29.95 | -9.633333 |
|  | DB201 | Clade 1 | 28.555337 | -10.150764 |
|  | DB202 | Clade 1 | 29.0169 | -10.0614 |
|  | DB203 | Clade 1 | 30.783 | -9.117 |
|  | DB205 | Clade 6 | 34.23687222 | -4.015147222 |
|  | DB206 | Clade 6 | 34.23687222 | -4.015147222 |
|  | DB207 | Clade 3 | 35.462695 | -6.027864 |
|  | DB208 | Clade 3 | 40.00426667 | 3.867725 |
|  | DB209 | Clade 3 | 40.00439444 | 3.867263889 |
|  | DB210 | Clade 3 | 40.00429167 | 3.867408333 |
|  | DB211 | Clade 2 | 37.09618611 | -3.39255 |
|  | DB214 | Clade 3 | 36.9 | 8.733333 |
|  | DB215 | Clade 3 | 36.9 | 8.733333 |
|  | DB216 | Clade 3 | 36.9 | 8.733333 |
|  | DB218 | Clade 3 | 36.9 | 8.733333 |
|  | DB265 | Clade 2 | 40.29892222 | -7.067225 |
|  | DB267 | Clade 3 | 35.88913611 | -5.343508333 |
|  | DB270 | Clade 3 | 35.94185833 | -3.033683333 |
|  | DB271 | Clade 3 | 36.74798056 | -3.023791667 |
|  | DB272 | Clade 3 | 36.773388 | -3.027842 |
|  | DB273 | Clade 3 | 35.30650833 | -2.954216667 |
|  | DB276 | Clade 2 | 40.99559722 | -3.235838889 |
|  | DB277 | Clade 3 | 37.58038333 | -6.914944444 |
|  | DB278 | Clade 3 | 37.3095 | -7.01525 |
|  | DB279 | Clade 3 | 36.376199 | -6.159044 |
|  | DB280 | Clade 2 | 37.67326111 | -2.965461111 |
|  | DB281 | Clade 3 | 36.91831944 | -3.480311111 |
|  | DB282 | Clade 2 | 38.75891111 | -2.449069444 |
|  | DB283 | Clade 2 | 38.18557222 | -1.890983333 |
|  | DB285 | Clade 2 | 36.887075 | -2.925619444 |
|  | DB286 | Clade 3 | 37.45917778 | -3.893913889 |
|  | DB287 | Clade 2 | 38.43013611 | -2.732766667 |
|  | DB288 | Clade 2 | 38.45315278 | -2.513702778 |
|  | DB289 | Clade 2 | 39.18496667 | -5.207416667 |
|  | DB291 | Clade 2 | 38.13350556 | -5.513063889 |
|  | DB292 | Clade 3 | 37.20356111 | -6.356680556 |
|  | DB293 | Clade 3 | 37.95843333 | -5.623255556 |
|  | DB294 | Clade 3 | 37.70200278 | -5.284316667 |
|  | DB295 | Clade 3 | 36.46815833 | -5.927766667 |
|  | DB296 | Clade 6 | 36.13963889 | -5.698825 |
|  | DB297 | Clade 3 | 36.6163 | -5.34497 |
|  | DB298 | Clade 3 | 36.209625 | -5.385469444 |
|  | DB311 | Clade 3 | 33.405433 | -5.103317 |
|  | DB313 | Clade 4 | 31.62292778 | -8.012647222 |
|  | DB314 | Clade 4 | 31.62292778 | -8.012647222 |
|  | DB317 | Clade 3 | 34.05 | -5.516667 |
|  | DB318 | Clade 6 | 32.67913056 | -4.738936111 |
|  | DB320 | Clade 1 | 31.51401944 | -9.762847222 |
|  | DB321 | Clade 1 | 31.51401944 | -9.762847222 |
|  | DB323 | Clade 3 | 34.87281667 | -6.294697222 |
|  | DB328 | Clade 3 | 34.87281667 | -6.294697222 |
|  | DB329 | Clade 6 | 33.50009722 | -3.820922222 |
|  | DB351 | Clade 3 | 37.156117 | 9.293383 |
|  | DB365 | Clade 3 | 36.372613 | -6.167205 |
|  | DB366 | Clade 2 | 37.74349722 | -2.551891667 |
|  | DB367 | Clade 2 | 37.74349722 | -2.551891667 |
|  | DB368 | Clade 2 | 37.74349722 | -2.551891667 |
|  | DB369 | Clade 3 | 36.372613 | -6.167205 |
|  | DB372 | Clade 2 | 41.27445833 | -7.397375 |
|  | DB373 | Clade 6 | 35.140483 | -5.134483 |
|  | DB374 | Clade 6 | 35.1569 | -5.1404 |
|  | DB376 | Clade 3 | 36.419153 | -6.149412 |
|  | DB377 | Clade 2 | 37.17706111 | -3.599436111 |
|  | DB379 | Clade 6 | 36.60495556 | -5.269069444 |
|  | DB380 | Clade 6 | 36.60495556 | -5.269069444 |
|  | DB381 | Clade 2 | 37.74349722 | -2.551891667 |
|  | DB382 | Clade 2 | 41.3505 | -7.29637 |
|  | DB383 | Clade 2 | 41.4038 | -6.36876 |
|  | DB393 | Clade 3 | 40.7113 | 0.50801 |
|  | DB394 | Clade 3 | 36.989759 | -6.443803 |
|  | DB395 | Clade 3 | 36.376199 | -6.159044 |
|  | DB398 | Clade 1 | 31.48441667 | -7.43175 |
|  | DB401 | Clade 6 | 36.95933 | -4.52994 |
|  | DB402 | Clade 3 | 37.139191 | -6.830254 |
|  | DB403 | Clade 3 | 37.45998 | -3.89826 |
|  | DB404 | Clade 3 | 37.4614 | -3.92858 |
|  | DB405 | Clade 3 | 38.01781 | -7.86429 |
|  | DB406 | Clade 3 | 37.24412995 | -6.381625112 |
|  | DB407 | Clade 2 | 38.18184 | -4.03798 |
|  | DB408 | Clade 3 | 37.15227979 | -6.7450643 |
|  | DB409 | Clade 3 | 36.682651 | -6.400441 |
|  | DB410 | Clade 3 | 36.508481 | -4.829723 |
|  | DB411 | Clade 2 | 39.96353333 | -3.504733333 |
|  | DB412 | Clade 2 | 39.44975 | -4.57413 |
|  | DB413 | Clade 2 | 38.22703 | -6.483 |
|  | DB414 | Clade 2 | 39.01987 | -6.17615 |
|  | DB415 | Clade 2 | 39.57593 | -4.57334 |
|  | DB416 | Clade 2 | 39.43269 | -7.57841 |
|  | DB417 | Clade 2 | 39.06119 | -3.00632 |
|  | DB420 | Clade 3 | 39.20042 | -2.16503 |
|  | DB423 | Clade 3 | 36.51154 | 9.07832 |
|  | DB424 | Clade 3 | 36.70873 | 9.18193 |
|  | DB425 | Clade 3 | 36.95954 | 8.75533 |
|  | DB426 | Clade 3 | 36.49269 | 8.57738 |
|  | DB474 | Clade 6 | 34.79708333 | 1.4296 |
|  | DB476 | Clade 3 | 36.56361667 | 4.051666667 |
|  | DB479 | Clade 3 | 36.47223333 | 4.007016667 |
|  | DB494 | Clade 3 | 36.40983333 | 2.753833333 |
|  | DB496 | Clade 3 | 36.44833333 | 4.125 |
|  | DB526 | Clade 3 | 40.5508 | 14.241 |
|  | DB687 | Clade 3 | 40.7493 | 14.0057 |
|  | DB688 | Clade 3 | 40.755509 | 14.015941 |
|  | DB696 | Clade 3 | 40.7514 | 14.0252 |
|  | DB703 | Clade 3 | 40.7153 | 13.9105 |
|  | DB720 | Clade 3 | 40.7434 | 13.9395 |
|  | DB721 | Clade 3 | 40.7434 | 13.9395 |
|  | DB722 | Clade 3 | 40.7072 | 13.9197 |
|  | DB724 | Clade 3 | 40.7072 | 13.9197 |
|  | DB756 | Clade 3 | 40.642181 | 14.448334 |
|  | DB766 | Clade 3 | 40.7514 | 14.0252 |
|  | DB780 | Clade 3 | 33.95625 | -6.85073 |
|  | DB911 | Clade 4 | 32.52575 | -7.86277 |
|  | DB924 | Clade 4 | 32.66101 | -7.79298 |
|  | DB925 | Clade 4 | 32.52575 | -7.86277 |
|  | DB933 | Clade 4 | 32.66101 | -7.79298 |
|  | DB934 | Clade 4 | 32.66101 | -7.79298 |
|  | DB935 | Clade 4 | 32.66101 | -7.79298 |
|  | DB1207 | Clade 3 | 40.59550333 | -6.53343 |
|  | DB1212 | Clade 3 | 40.59550333 | -6.53343 |
|  | DB1236 | Clade 2 | 38.10894667 | -1.860658333 |
|  | DB1246 | Clade 3 | 38.31501167 | -4.625581667 |
|  | DB1293 | Clade 2 | 38.33816 | -1.65265 |
|  | DB1294 | Clade 2 | 38.49741833 | -2.434656667 |
|  | DB1295 | Clade 2 | 38.52175667 | -2.346721667 |
|  | DB1397 | Clade 1 | 31.74957833 | -8.738441667 |
|  | DB1400 | Clade 1 | 31.74957833 | -8.738441667 |
|  | DB1448 | Clade 1 | 30.83131944 | -8.861022222 |
|  | DB1519 | Clade 3 | 35.46904833 | -6.025656667 |
|  | DB1571 | Clade 3 | 36.249065 | 10.424635 |
|  | DB1666 | Clade 3 | 43.608176 | 3.879446 |
|  | DB1667 | Clade 3 | 43.608176 | 3.879446 |
|  | DB1774 | Clade 2 | 38.37168167 | -2.578653333 |
|  | DB1786 | Clade 2 | 38.28569 | -2.6485 |
|  | DB1801 | Clade 2 | 38.29546667 | -2.614755 |
|  | DB1880 | Clade 2 | 38.997652 | -1.86007 |
|  | DB2545 | Clade 1 | 30.82166667 | -8.856111111 |
|  | DB2563 | Clade 4 | 32.49303 | -7.90857 |
|  | DB2635 | Clade 1 | 30.26 | -8.75 |
|  | DB2636 | Clade 1 | 30.776675 | -8.856647222 |
|  | DB2818 | Clade 2 | 38.323485 | -2.633985 |
|  | DB2886 | Clade 2 | 38.37168167 | -2.578653333 |
|  | DB3131 | Clade 3 | 41.98408064 | 12.05039101 |
|  | DB3142 | Clade 3 | 40.65030248 | 17.1120226 |
|  | DB3144 | Clade 3 | 40.65030248 | 17.1120226 |
|  | DB3145 | Clade 3 | 40.7853202 | 17.20584032 |
|  | DB3146 | Clade 3 | 40.65030248 | 17.1120226 |
|  | DB3148 | Clade 3 | 40.65030248 | 17.1120226 |
|  | DB3152 | Clade 3 | 39.2724182 | 9.311272539 |
|  | DB3170 | Clade 3 | 41.74677405 | 12.36981473 |
|  | DB3176 | Clade 3 | 41.74677405 | 12.36981473 |
|  | DB3179 | Clade 3 | 41.74677405 | 12.36981473 |
|  | DB3180 | Clade 3 | 40.65030248 | 17.1120226 |
|  | DB3182 | Clade 3 | 39.73202938 | 9.020935338 |
|  | DB3231 | Clade 3 | 44.114878 | 15.228951 |
|  | DB3232 | Clade 3 | 44.114878 | 15.228951 |
|  | DB3233 | Clade 3 | 43.172591 | 16.44323 |
|  | DB3234 | Clade 3 | 44.114878 | 15.228951 |
|  | DB3235 | Clade 3 | 43.172591 | 16.44323 |
|  | DB3236 | Clade 3 | 43.172591 | 16.44323 |
|  | DB3238 | Clade 3 | 44.114878 | 15.228951 |
|  | DB3239 | Clade 3 | 43.172591 | 16.44323 |
|  | DB3832 | Clade 2 | 37.666667 | -0.75 |
|  | DB3834 | Clade 2 | 37.696002 | -0.771109 |
|  | DB3838 | Clade 2 | 37.727633 | -0.707559 |
|  | DB3839 | Clade 2 | 37.727633 | -0.707559 |
|  | DB3843 | Clade 2 | 37.666667 | -0.75 |
|  | DB3844 | Clade 2 | 37.666667 | -0.75 |
|  | DB3846 | Clade 2 | 37.666667 | -0.75 |
|  | DB3847 | Clade 2 | 38.166667 | -0.466667 |
|  | DB3848 | Clade 2 | 38.166667 | -0.466667 |
|  | DB3853 | Clade 2 | 37.609237 | -0.993119 |
|  | DB5057 | Clade 4 | 32.43988 | -5.98789 |
|  | DB5065 | Clade 6 | 33.64037 | -4.2007 |
|  | DB5113 | Clade 6 | 33.64037 | -4.2007 |
|  | DB5142 | Clade 3 | 40.7113 | 0.50801 |
|  | DB9076 | Clade 4 | 31.8894323 | -7.942285746 |
|  | DB9104 | Clade 3 | 38.813055 | 16.25749833 |
|  | DB9107 | Clade 3 | 39.017407 | 17.138991 |
|  | DB9111 | Clade 3 | 38.700273 | 15.991034 |
|  | DB9112 | Clade 3 | 40.65040667 | 14.63969333 |
|  | DB9113 | Clade 3 | 38.70896 | 15.97311167 |
|  | DB9115 | Clade 3 | 38.813055 | 16.25749833 |
|  | DB9116 | Clade 3 | 39.970982 | 16.595627 |
|  | DB11003 | Clade 1 | 31.6494 | -8.01577 |
|  | DB11004 | Clade 1 | 31.6494 | -8.01577 |
|  | DB11007 | Clade 1 | 31.57595 | -9.014601 |
|  | DB11008 | Clade 1 | 31.6494 | -8.01577 |
|  | DB11009 | Clade 1 | 30.75148 | -7.2979 |
|  | DB11011 | Clade 3 | 40.65040667 | 14.63969333 |
|  | DB11013 | Clade 1 | 31.6494 | -8.01577 |
|  | DB11015 | Clade 4 | 31.8894323 | -8.013233 |
|  | DB11019 | Clade 4 | 31.8894323 | -8.013233 |
|  | DB11021 | Clade 4 | 31.8894323 | -7.942285746 |
|  | DB11022 | Clade 1 | 31.67747 | -9.61136 |
|  | DB11029 | Clade 4 | 31.8894323 | -8.013233 |
|  | DB11035 | Clade 1 | 31.57595 | -9.014601 |
|  | DB11042 | Clade 1 | 31.6494 | -8.01577 |
|  | DB11091 | Clade 3 | 42.30896 | 9.14597 |
|  | DB11097 | Clade 3 | 42.45012 | 9.2325 |
|  | DB11100 | Clade 3 | 40.163878 | 18.007209 |
|  | DB11105 | Clade 3 | 40.18515 | 17.92660333 |
|  | DB3087 | Clade 6 | 34.90009 | -3.59291 |
|  | DB3265 | Clade 6 | 32.83479 | -4.3283 |
